# Deep learning-driven TCR$\beta$ repertoire analysis enhances diagnosis and enables mining of immunological biomarkers in systemic lupus erythematosus

**DOI:** 10.1186/s13040-025-00490-5

**Published:** 2025-10-31

**Authors:** Tongfei Shen, Yifei Sheng, Wan Nie, Shuo Yang, Kaiqi Li, Ziwei Ma, Zhao Ling, Bowen Tan, Xikang Feng, Miaozhe Huo

**Affiliations:** 1 Department of Computer Science, City University of Hong Kong, Kowloon, Hong Kong China; 2 School of Software, Northwestern Polytechnical University, Xi’an, Shaanxi China

**Keywords:** Systemic lupus erythematosus, Deep learning, TCR$\beta$ CDR3 sequence, Diagnosis of autoimmune diseases

## Abstract

**Background:**

Systemic Lupus Erythematosus (SLE) is a complex autoimmune disorder involving dysregulation of multiple immune components, including T cells. Aberrant T-cell activity contributes significantly to the immune pathology of SLE, for instance, by facilitating autoantibody production. The Complementarity Determining Region 3 (CDR3) of the TCR$\beta$ chain is pivotal for T-cell specificity, thereby positioning it as a promising target for enhancing diagnostic accuracy and gaining deeper mechanistic insights into SLE. To address these diagnostic limitations in SLE, our team developed DeepTAPE, a deep learning-based diagnostic framework that utilizes CDR3 sequences to achieve robust classification performance for SLE.

**Results:**

Building upon the foundation established by DeepTAPE, we devised a novel diagnostic approach that effectively integrates a TCR classifier to quantify SLE disease activity. Furthermore, this methodology employs advanced deep learning models for the bio-mining of disease-associated motifs that serve as potential biomarkers. As a result, this approach generates an autoimmune risk score (ARS) indicative of SLE probability. Notably, this ARS metric exhibited a strong correlation with disease activity, functioning as a quantitative clinical marker that complements traditional indices such as the SLE Disease Activity Index (SLEDAI). In addition, through a comprehensive analysis of immune repertoire data, we identified SLE-specific amino acid motifs within the CDR3 sequences, including critical 3-mer and gapped-mer oligopeptides. These motifs demonstrated high efficacy in SLE classification, achieving an area under the curve (AUC) of 0.908, thereby significantly outperforming other candidate biomarkers. Moreover, our model revealed potential SLE-associated antigens and genes, such as *CD109* and *INS*, which provide new insights into the immunological mechanisms underlying the disease.

**Conclusion:**

This study highlights the potential of DeepTAPE as a supportive tool for biomarker discovery and assessing SLE disease activity, which complements traditional diagnostic approaches. By deepening our understanding of the immunological characteristics and mechanisms associated with SLE, this work lays a foundation for advancing targeted therapies and personalized medicine in autoimmune diseases. Consequently, our findings may pave the way for improved patient outcomes and more effective treatment strategies in the management of SLE.

**Supplementary Information:**

The online version contains supplementary material available at 10.1186/s13040-025-00490-5.

## Introduction

Systemic lupus erythematosus (SLE) is a chronic autoimmune disorder characterized by substantial morbidity, affecting multiple organs and systems [1–4]. The pathogenesis of SLE involves a complex interplay of genetic, environmental, and hormonal factors, which collectively lead to autoantibody overproduction, immune complex formation, and T cell infiltration, ultimately culminating in tissue damage [5–10]. Furthermore, the diagnostic criteria established by the American College of Rheumatology integrate clinical, laboratory, and imaging assessments; however, these criteria exhibit significant limitations in precision and timeliness, alongside a notable lack of practical tools for effective disease activity monitoring [11–16].

In recent years, high-throughput sequencing technologies in immunomics have significantly advanced diagnostic capabilities by enabling a comprehensive analysis of the immune repertoire [17–19]. Among these advancements, the T cell receptor (TCR), a transmembrane protein located on T cell surfaces, has emerged as a key focus due to its remarkable diversity arising from genetic rearrangement and somatic mutation, which facilitates effective antigen recognition [20]. Notably, the TCR $\beta$ chain, particularly the third complementarity-determining region (CDR3), is highly diverse and critical for antigen binding [21–28]. Multiple studies have demonstrated that TCR$\beta$ diversity profoundly influences autoimmune responses in systemic lupus erythematosus (SLE) and rheumatoid arthritis, where distinct V, J, and V–J gene pairings serve as diagnostic biomarkers [29–33]. Bioinformatics analyses supported by clinical data indicate that alterations in the TCR$\beta$ clonal architecture represent robust diagnostic markers for SLE. A recent 2025 study identified nine hub TRBV genes with significantly elevated expression (AUC 0.985$-$1.000) that effectively distinguish SLE patients from healthy controls, and reported a negative correlation between TCR$\beta$ diversity (D50) and disease activity [33]. Additionally, oligoclonal expansions of TCR V$\beta$ families have been associated with increased SLE severity, underscoring their potential for non-invasive molecular diagnosis [30]. As a key component of SLE’s highly heterogeneous immune dysregulation, T cell dysfunction contributes significantly to its pathogenesis, even if it is not the sole or defining pathological hallmark of the disease [34, 35]. The TCR CDR3 sequence reflects the state of the T cell repertoire, and its analysis captures disease-associated immune repertoire changes. While CDR3 sequence features do not directly prove T cell dysfunction as the causal factor, their use as auxiliary diagnostic biomarkers for SLE primarily stems from their ability to provide a unique perspective on adaptive immune status [20].

Leveraging deep learning techniques, related studies have demonstrated significant promise in immune repertoire-based diagnostics. For instance, Xu et al. demonstrated the potential of this strategy in oncology by developing DeepLION, a convolutional neural network (CNN) model that classifies thyroid cancer cohorts from TCR$\beta$ CDR3 sequences with an AUC of 0.90 [36]. Further demonstrating this strategy’s potential in autoimmunity, Rawat et al. used the DeepRC model to classify type 1 diabetes from TCR$\beta$ repertoires. Their approach not only achieved an AUC of 0.77 but also successfully identified disease-relevant sequence features [37]. These successes underscore the promise of applying deep learning to TCR$\beta$ CDR3 sequence patterns, which could revolutionize SLE classification [32, 38].

In addition, immunogenomic approaches combined with machine learning, such as random forest classifiers, leverage V, D, and J gene frequency distributions in TCR $\beta$ as diagnostic markers for SLE [31]. However, it is crucial to note that the focus of this method on gene distribution overlooks critical sequence-level information, thereby limiting biomarker identification and the quantification of SLE disease activity. In response to these challenges, our previously developed DeepTAPE framework addresses these limitations by utilizing CDR3 sequences and V gene features through a CNN-LSTM architecture with residual connections, achieving an impressive 97.99% AUC and 93.97% accuracy [39]. Nevertheless, key challenges persist in objectively quantifying disease severity and identifying biomarkers with clinical utility.

Moreover, when considering the quantification of SLE activity, Bombardier et al. pioneered the SLE Disease Activity Index (SLEDAI), which systematically quantifies SLE activity through 24 weighted clinical and laboratory parameters, including arthritis, rash, proteinuria, and complement levels [40]. This groundbreaking tool has since established itself as the foundational standard for SLE clinical trials and management. Subsequently, Gladman et al. introduced SLEDAI-2K as an optimized version, extending the evaluation window from 10 to 30 days and modifying criteria for persistent manifestations, such as proteinuria and mucocutaneous lesions [41]. However, challenges persist, including temporal constraints, clinician dependency, laboratory burden, and threshold ambiguity, which continue to overshadow the utility of SLEDAI. These limitations underscore the urgent need for further refinement and innovation in SLE disease activity assessment tools. For instance, Ergun et al. attempted to use the Systemic Immune-Inflammation Index (SII) for SLE monitoring but achieved limited accuracy (AUC 0.678) due to small sample sizes [42]. The SII is calculated as (platelet count $\times$ neutrophil count)/lymphocyte count and represents a composite inflammatory marker that integrates information from three different immune cell populations to assess systemic inflammation status. Given these hurdles, it is essential that we develop more effective approaches.

In this study, we address the existing challenges by presenting several key advancements in the understanding and diagnosis of autoimmune diseases. Specifically, we introduce an autoimmune risk score (ARS) derived from deep learning analysis of TCR$\beta$ CDR3 sequences. In contrast to conventional SLE assessment standards like SLEDAI, which rely on multiple biochemical tests and the physician’s subjective judgment [40, 43], our data-driven approach offers a more direct and objective evaluation. By leveraging the well-trained deepTAPE model, we identify SLE-related TCR sequences with the highest inference probabilities within the population’s TCR repertoire and subsequently map these sequences to candidate antigens, antibodies, and genes implicated in autoimmune pathology. Furthermore, salience analysis of these candidate sequences enables the identification of specific oligopeptide motifs that may serve as potential biomarkers for SLE, thereby providing new evidence for our understanding of its immunological characteristics and pathogenesis. In summary, our findings offer quantitative indicators for SLE activity and severity, presenting an alternative framework that complements traditional assessments with data-driven insights while facilitating the identification of biomarkers from patient TCR repertoire data.

## Methods

### Framework overview

**Fig. 1:**
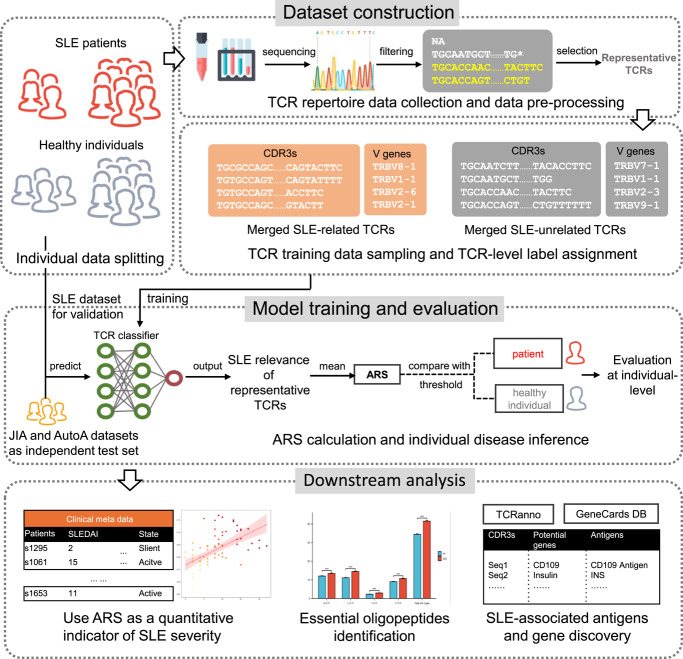
Workflow of the DeepTAPE framework. The pipeline diagram outlines three main modules: Dataset Construction, Model Training and Evaluation, and Downstream Analysis. Initially, equal numbers of systemic lupus erythematosus (SLE) patients and healthy individuals are randomly selected, and peripheral blood mononuclear cell (PBMC)-derived TCR data are filtered and sorted by clone type frequency to create representative TCR sets for training. During the Model Training and Evaluation phase, sequences and gene features are standardized through zero-padding, tokenized, and fed into the T cell receptor (TCR) classifier. The classifier calculates Autoimmune Risk Score (ARS) to distinguish between patients and healthy individuals, validated via cross-validation and independent tests. Downstream analysis involves exploring correlations of ARS with disease activity indices, identifying essential oligopeptides, and discovering autoimmune-associated antigens and genes among SLE patients

Our analytical framework is structured around three principal stages: (i) dataset construction, (ii) model training and evaluation, and (iii) downstream analysis. This pipeline was designed to identify SLE-related TCR signatures from repertoire data and use them to develop a quantitative indicator named Autoimmune Risk Score (ARS). A schematic of this process is presented in Fig. 1.

### Data collection

The main dataset for this study, comprising TCR sequences from 439 healthy individuals and 877 SLE patients [31], was sourced from publicly accessible databases and includes additional disease activity data for quantitative analysis. All SLE patients fulfilled the American College of Rheumatology classification criteria. For rigorous model assessment, we incorporated several external cohorts, including samples from patients with juvenile idiopathic arthritis and autoimmune arthritis [44, 45], along with healthy controls from another study [46]. These external cohorts collectively formed the independent test set for this study. For further details on all data sources, please refer to Supplementary 1.1.

### Dataset construction

#### Main datasets

For each repertoire from the main dataset [31], we first derived a set of representative TCR clonotypes. This involved an initial quality control filtering step followed by the selection of the 2,000 most frequent clonotypes. This data pre-processing was applied uniformly across all repertoires to ensure methodological consistency.

Following this TCR clonotypes processing, the entire cohort of individuals was partitioned into a training set and a validation set at a 4:1 ratio. The split was performed at the individual level to prevent data leakage. The representative TCRs from all individuals in the training cohort were then pooled to construct the final TCR-level database used for developing the DeepTAPE TCR classifier. From this database, we generated three distinct feature sets to train our models: (1) CDR3 sequences alone, (2) CDR3 sequences paired with specific V-gene variants, and (3) CDR3 sequences paired with their V-gene family.

The validation set, comprising repertoires from individuals entirely unseen during training, was reserved for assessing the DeepTAPE SLE classification utility at the individual-level. Specifically, the representative TCRs from these subjects were used as input to the trained classifier to infer an Autoimmune Risk Score (ARS). Further details on these procedures are provided in Supplementary 1.2.

#### Independent testset

To ensure a fair and unbiased external validation, all repertoires in the independent test set were subjected to the identical quality control and frequency-based selection criteria used for the main dataset. This guarantees consistent data handling prior to model inference.

### Model architecture

**Fig. 2:**
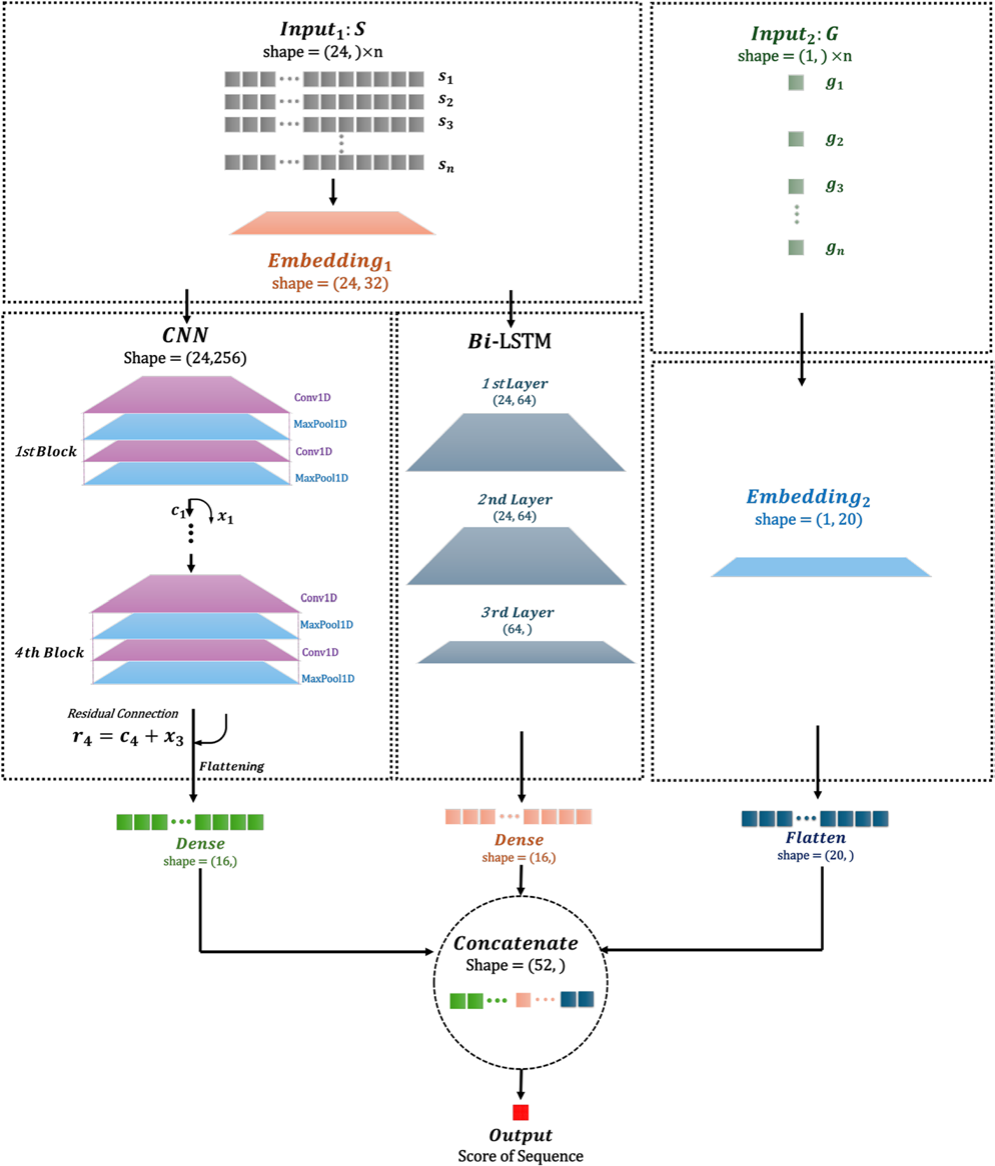
TCR classifier model architecture. DeepTAPE classifier integrates CNN and LSTM to analyze two types of inputs: sequence set $S$ and gene set $G$. After embedding, $S$ is processed by the CNN, featuring four convolutional blocks with residual connections, and by the LSTM, comprising three bidirectional layers with dropout. $G$ is embedded and flattened before being concatenated with the outputs from the CNN and LSTM. This concatenated mix is then advanced through a fully connected layer to generate the final prediction score for the input TCR data

The model architecture integrates Convolutional Neural Networks and Long Short-Term Memory layers, utilizing three datasets with different feature combinations to classify TCR sequences (Fig. 2). Detailed design, optimization, and formulas are provided in Supplementary 1.3.

### Model training and evaluation

The DeepTAPE classifier was trained on the TCR-level database derived from the training set, framed as a binary classification task to distinguish SLE-related from non-SLE-related TCRs. We then rigorously evaluated the model’s clinical utility at the individual level using a 5-fold cross-validation strategy on the held-out validation set. This evaluation assessed the capacity of the inferred Autoimmune Risk Score (ARS) to differentiate autoimmune patients from healthy controls. The generalizability of our final framework was further evaluated by its performance on the independent test sets. A comprehensive description of training hyperparameters, evaluation metrics, and comparisons with baseline models is provided in Supplementary 1.4 and 1.5.

### Quantitative evaluation of the ARS as an indicator of SLE disease activity

The ARS was computed from representative TCR sequences to indicate the probability of SLE, and its performance metrics were assessed to ensure accurate quantification of disease activity. In our study, we also investigated the relationship between ARS and other traditional clinical indicators related to disease activity, supplemented by correlation analysis.

- *SLEDAI* is a standard score of SLE activity calculated by adding weighted clinical and laboratory items from the past 10 days; a higher score indicates more active disease. (Supplementary Fig. S4)
- *SLE Disease Activity Status* was determined based on SLEDAI cutoffs following established clinical guidelines [41]. Patients with SLEDAI scores $\ge$ 5 were classified as having “Active” disease, while those with SLEDAI scores < 5 were classified as “Silent” (inactive disease), shown in Supplementary Table S4. This threshold is widely accepted in clinical practice for distinguishing between active and inactive SLE disease states [47].
- C3 is a major complement protein produced in the liver; in active SLE, it is consumed by immune-complex activation, so blood C3 levels are low and tend to decrease as disease activity increases.
- C4 is a classical-pathway complement protein also produced in the liver; active SLE consumes C4 as well, so low C4 often indicates higher activity, especially in lupus nephritis.
- *Anti-dsDNA antibodies* are autoantibodies against double-stranded DNA produced by autoreactive B cells; higher levels usually correlate with increased SLE activity, particularly kidney involvement.
- *Clinical damage* in our study refers to irreversible organ or tissue injury accumulated over the course of SLE progression, representing permanent structural or functional impairment to various organ systems caused by SLE disease activity, treatment complications, or comorbidities. Unlike disease activity measures such as SLEDAI, which reflect current inflammatory processes that may be reversible, damage represents cumulative, irreversible pathological changes that persist regardless of current disease activity status. Based on our analysis of the supplementary data, we identified five distinct types of damaged tissues documented in our patient cohort: skin, joint, blood, kidney, and brain. The damage assessment categorizes patients based on the number of different tissue types or organ systems that have sustained permanent damage, ranging from “None” (no documented damage) to “Four” (four different organ/tissue types affected).

To validate the utility of the ARS, we employed one-way ANOVA to compare score distributions between different patient groups and healthy controls. Furthermore, the association between ARS and established disease activity markers, including SLEDAI, C3, and C4 levels, was quantified using both Pearson’s correlation coefficient to assess linear relationships and Spearman’s rank correlation coefficient to evaluate monotonic trends. Detailed descriptions of these statistical methods and their corresponding formulas are available in Supplementary Section 1.7.

### Identifying essential 3-mer oligopeptides as potential SLE biomarkers

TCR-pMHC crystal structure analyses demonstrate that motifs within the CDR3$\beta$ loop help to form the functional core of molecular recognition [48]. Therefore, identifying the essential 3-mer oligopeptides that contribute most to an SLE-associated TCR holds the potential to reveal novel biomarkers for the disease. In this study, the top 2,000 highest-scoring sequences from SLE patient samples were selected for saliency analysis. For each sequence, we applied a masking technique where each 3-mer oligopeptide was replaced by three consecutive zeros, thereby nullifying the corresponding amino acids. This approach allowed us to perform a saliency analysis to assess the impact of every 3-mer oligopeptide on the DeepTAPE.

We compared the predictive probabilities of the masked sequences to those of the original sequences. The difference in scores was calculated to quantify the contribution of each 3-mer oligopeptide to the SLE relevance of the whole CDR3 sequence. 3-mer oligopeptides with high scores (in the top 1.5% of scores) and occurring with a minimum frequency of 300 were identified as potential essential oligopeptides. To further validate their significance, we analyzed the frequency of these essential 3-mer oligopeptides in both SLE and healthy individual samples, looking for statistically significant differences as determined by an independent t-test. The classification performance of these oligopeptides was assessed using the AUC metric to distinguish between SLE patients and HIs. To investigate the positional distribution of scoring contributions from 3-mer oligopeptides within CDR3 amino acid sequences, we implemented a multi-faceted analytical approach. First, a heatmap was generated to visualize the score distribution of 3-mer oligopeptides across sequence positions, excluding zero-padding regions at the start, thereby identifying enrichment zones of high-scoring 3-mers. Second, sequences were partitioned into front, mid, and tail thirds to quantify the proportional differences of high-scoring, high-frequency 3-mers among segments. Third, clustered bar charts were employed to assess positional score variations of specific high-scoring, high-frequency 3-mers across segments. Additionally, the potential bias in TRBV and TRBJ usage associated with sequences containing essential 3-mer oligopeptides is discussed in detail in Supplementary Section 2.3.

### Identifying essential gapped mer oligopeptides as potential SLE biomarkers

Contiguous 3-mers are limited to capturing strictly local sequence similarity within the CDR3 region. We therefore extended our analysis to identify gapped mers, as they can capture essential motifs that tolerate single amino acid variations, reflecting a global sequence similarity [49]. The methodology for identifying these gapped mers was analogous to the 3-mer analysis, involving a masking technique where specific amino acids in a sequence are replaced with zeros. In this case, the masked elements were gapped mer oligopeptides of the form $X*XX$ and $XX*X$, where $X$ represents the amino acids being evaluated and $*$ indicates a position that remains unchanged.

We aimed to compare the predictive probabilities of the masked sequences with those of the original sequences. The difference in scores was calculated to quantify the contribution of each gapped mer oligopeptide to the SLE relevance of the entire CDR3 sequence. Specifically, for the gapped mer oligopeptides $X*XX$ and $XX*X$, those with high scores (within the top 1.5%) and occurring with a minimum frequency of 20 were identified as potential gapped mers. The frequency threshold was lowered due to the more dispersed nature of gapped mers, resulting in relatively lower frequencies.

To further validate their significance, we analyzed the frequency of these essential gapped mer oligopeptides in both SLE and healthy individual samples, seeking statistically significant differences as determined by an independent t-test. The classification performance of these oligopeptides was assessed using the AUC metric to distinguish between SLE patients and healthy individuals. This approach is analogous to the identification of 3-mer oligopeptides.

We must return to the fundamental goal of identifying biomarkers. Through the previous steps, we have identified essential 3-mer oligopeptides and gapped mer oligopeptides. We will examine the frequency differences of these biomarkers between the TCR repertoires of SLE and HI using the AUC curve. Additionally, we will compare these findings with potential existing initial screening diagnostic indicators, such as the SII, to validate their diagnostic effectiveness.

### Identifying potential antigens and genes related to SLE

Although the hallmark autoantigens initiating SLE are nucleic acid-protein complexes [50], the subsequent evolution and broadening of the autoimmune repertoire critically depend on T-cells recognizing specific peptide epitopes. Our study, therefore, focuses on identifying these key epitopes involved in the T-cell-mediated response. Such epitopes may be derived directly from the proteolytic processing of the initial autoantigen complexes or emerge later through mechanisms such as epitope spreading [51] and TCR cross-reactivity [52]. To elucidate potential antigens and associated genes implicated in SLE, we initiated our investigation by selecting the highest-scoring 2,000 sequences from the SLE patients in the third fold of the DeepTAPE validation set. This curated dataset of TCR sequences is hypothesized to contain paratopes likely to interact with SLE-related antigens. To identify these interactions, we utilized the TCRanno package [53] to map the selected TCR sequences to their corresponding epitopes. This analysis enabled us to identify SLE-associated antigens, along with the corresponding genes. From all candidate antigens, we retained those derived from Homo sapiens, aiming to identify antigens potentially related to autoimmune diseases.

We further used *InnateDB* [54] to identify antigen-related genes, then *GeneCards* [55] to extract autoimmune-associated pathologies. A meticulous review of the relevant academic literature was undertaken to refine our selection criteria, ensuring the inclusion of antigens substantiated as pertinent to autoimmune disorders. This multi-step screening process allowed us to identify candidates with high confidence for antigens and genes related to the pathogenesis of SLE.

## Results

### Assessing ARS as an immunological biomarker for quantifying SLE disease severity

Previous studies have validated the classification performance of DeepTAPE, demonstrating its effective prediction of SLE and related diseases. The highest cross-validated AUC for SLE reached 97.99% ± 0.82%, and the model generalized well to related autoimmune diseases with an independent external test AUC of 95.78% ± 0.19%, outperforming baselines composed of CNN, CNN-LSTM, Bi-LSTM, and SimpleRNN (Supplementary Sections 1.5, 1.6, 2.1, and 2.2). Detailed results are available in the supplementary materials (Supplemental Tables 1 and 2). Specifically, features derived from the TCR$\beta$ repertoire of SLE patients, such as CDR3 amino acid sequences and V-gene usage, can be utilized to assess disease activity levels. For further details, please refer to Supplementary 2.1 and 2.2. Thus, the embedded information within these amino acid sequences and gene frequencies from patients diagnosed with SLE can serve as valuable indicators for evaluating disease activity.

**Fig. 3:**
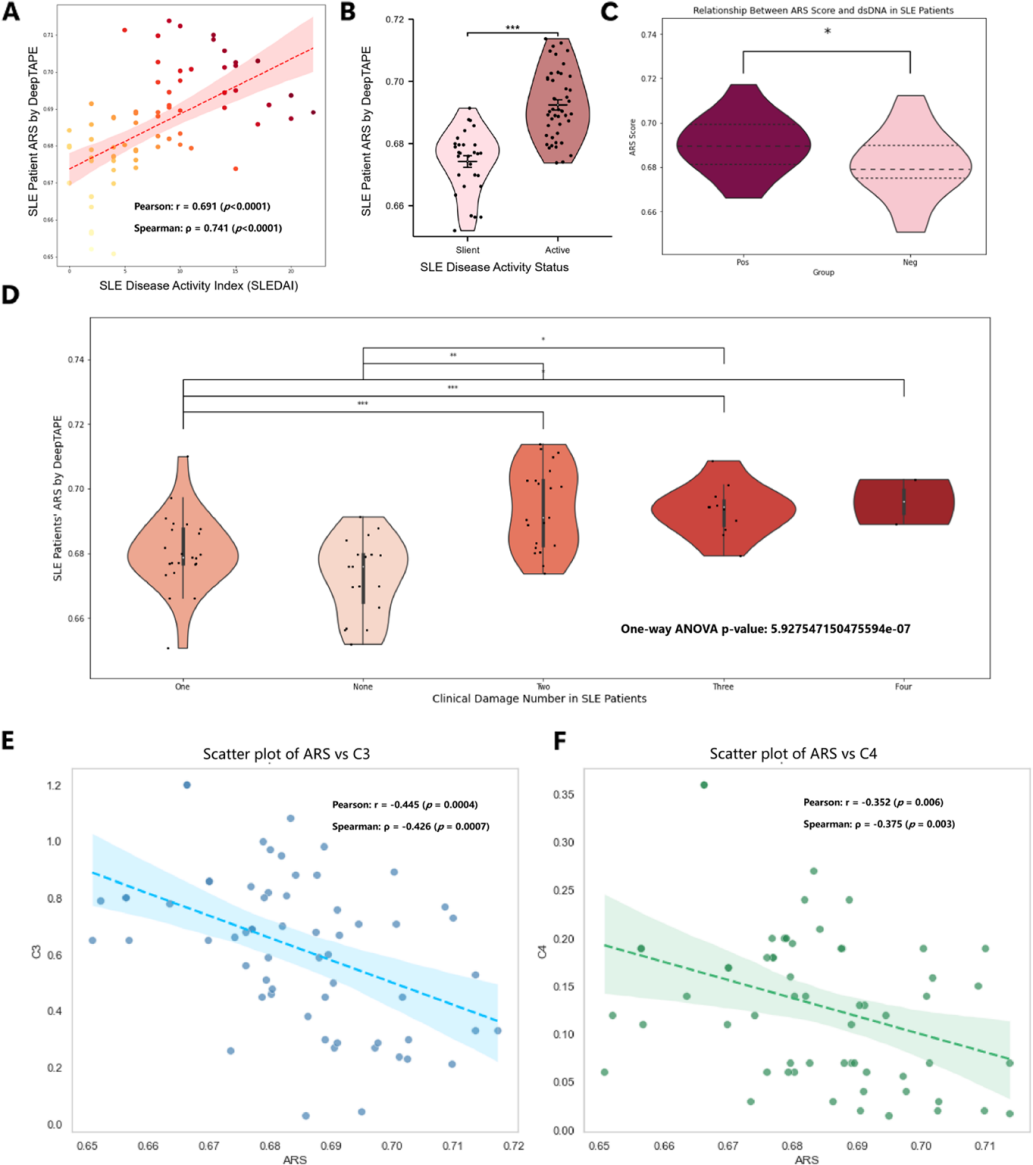
Association between the ARS derived from the TCR$\beta$ CDR3 based on a deep learning model and clinical disease activity. **A** Scatter plot with fitted regression line showing a significant positive correlation between ARS and clinical SLEDAI, confirmed by both Pearson and Spearman correlation coefficients. **B** Violin plot illustrating that ARS is significantly higher in patients with active disease compared to those in a silent state. **C** ARS is elevated in dsDNA antibody-positive patients relative to negatives. **D** ARS increases with the number of clinical damages among SLE patients, as determined by One-way ANOVA with Tukey’s post-hoc test. **E** Scatter plot showing a significant negative correlation between ARS and complement C3, supported by both Pearson and Spearman correlation coefficients. **F** Scatter plot illustrating a significant negative correlation between ARS and complement C4, confirmed by Pearson and Spearman correlation coefficients. *$p < 0.05$, **$p < 0.01$, and ***$p < 0.001$

In this context, a comprehensive analysis of the clinical data associated with the third fold of the DeepTAPE test set reveals a positive correlation between the autoimmune risk score (ARS) projected by the DeepTAPE model and the clinical assessment represented by the Systemic Lupus Erythematosus Disease Activity Index (SLEDAI). Specifically, the Pearson correlation coefficient is $r = 0.691$ ($p < 0.0001$), indicating a strong and highly significant linear positive correlation. Additionally, the Spearman correlation coefficient is $\rho = 0.741$ ($p < 0.0001$), which further supports a significant monotonic positive relationship. Both correlation measures are consistent, demonstrating that ARS and clinical SLEDAI exhibit a robust, reliable, and statistically significant positive association (Fig. 3A). Furthermore, an examination of the SLE disease activity status, as determined clinically, indicates that patients categorized in the “Active” state exhibit a significantly higher average ARS, as projected by the DeepTAPE model, compared to those classified in the “Silent” state (independent samples t-test, $p < 0.001$, Fig. 3B).

Turning to another traditional clinical biomarker for disease activity, anti-double-stranded DNA antibodies (dsDNA) are one of the specific autoantibodies in SLE, particularly relevant during disease flares. The presence of dsDNA antibodies aids in confirming the diagnosis of SLE and serves as a biomarker for disease activity. Here, patients positive for dsDNA antibodies show a significantly higher average ARS than those negative (independent samples t-test, $p < 0.05$), further supporting the reliability of ARS in assessing clinical SLE disease activity (Fig. 3C). These findings strongly suggest that the deep learning model, which incorporates TCR$\beta$ CDR3 sequences and their associated V-gene information as input features, provides a prediction of the patient’s disease activity level that closely aligns with clinical evaluations. Consequently, this reinforces the diagnostic utility of TCR $\beta$ CDR3 in the context of autoimmune diseases.

We further investigated whether the ARS, derived from the TCR $\beta$ CDR3 deep learning model, correlates with the extent of clinical damage in patients. In this context, clinical damage was defined as irreversible organ or tissue injury accumulated during SLE progression, with our cohort exhibiting damage across five systems: skin, joint, blood, kidney, and brain. We observed a positive association, where the average ARS tended to increase as the number of affected organ systems rose from none to four. Statistical analysis supported this observation. A one-way ANOVA indicated a significant overall difference in ARS across the damage categories ($p < 0.001$). Furthermore, Tukey’s post-hoc test confirmed that most pairwise comparisons between the damage groups were significant ($p < 0.05$). Nevertheless, statistical significance was not achieved for all comparisons, particularly between adjacent damage groups (Fig. 3D). Taken together, these results suggest that the ARS reflects the burden of cumulative clinical damage. However, the model’s current resolution may be insufficient to reliably differentiate between each incremental step of organ system involvement.

In addition, ARS shows consistent associations with two other conventional clinical indicators. Complement components C3 and C4, which are well-established biomarkers of SLE disease activity, typically display low serum levels during active disease phases, reflecting immune system overactivation and heightened inflammation. Our study reveals a significant negative correlation between ARS and complement C3, with Pearson’s correlation coefficient $r = -0.445$ ($p = 0.0004$) and Spearman’s correlation coefficient $\rho = -0.426$ ($p = 0.0007$), demonstrating a moderate and highly significant linear and monotonic negative correlation, respectively (Fig. 3E). Similarly, ARS is significantly negatively correlated with complement C4, with Pearson’s $r = -0.352$ ($p = 0.006$) and Spearman’s $\rho = -0.375$ ($p = 0.003$), both indicating statistically significant negative correlations (Fig. 3F). Since low complement C3 and C4 levels indicate active or exacerbated SLE, the elevated ARS scores observed correspond well with these traditional clinical markers. This concordance underscores the predictive value of ARS for SLE disease progression and highlights its reliability as an immunological biomarker consistent with established clinical indices.

### Role of model-identified 3-mer oligopeptides in CDR3 for autoimmune disease classification

**Fig. 4:**
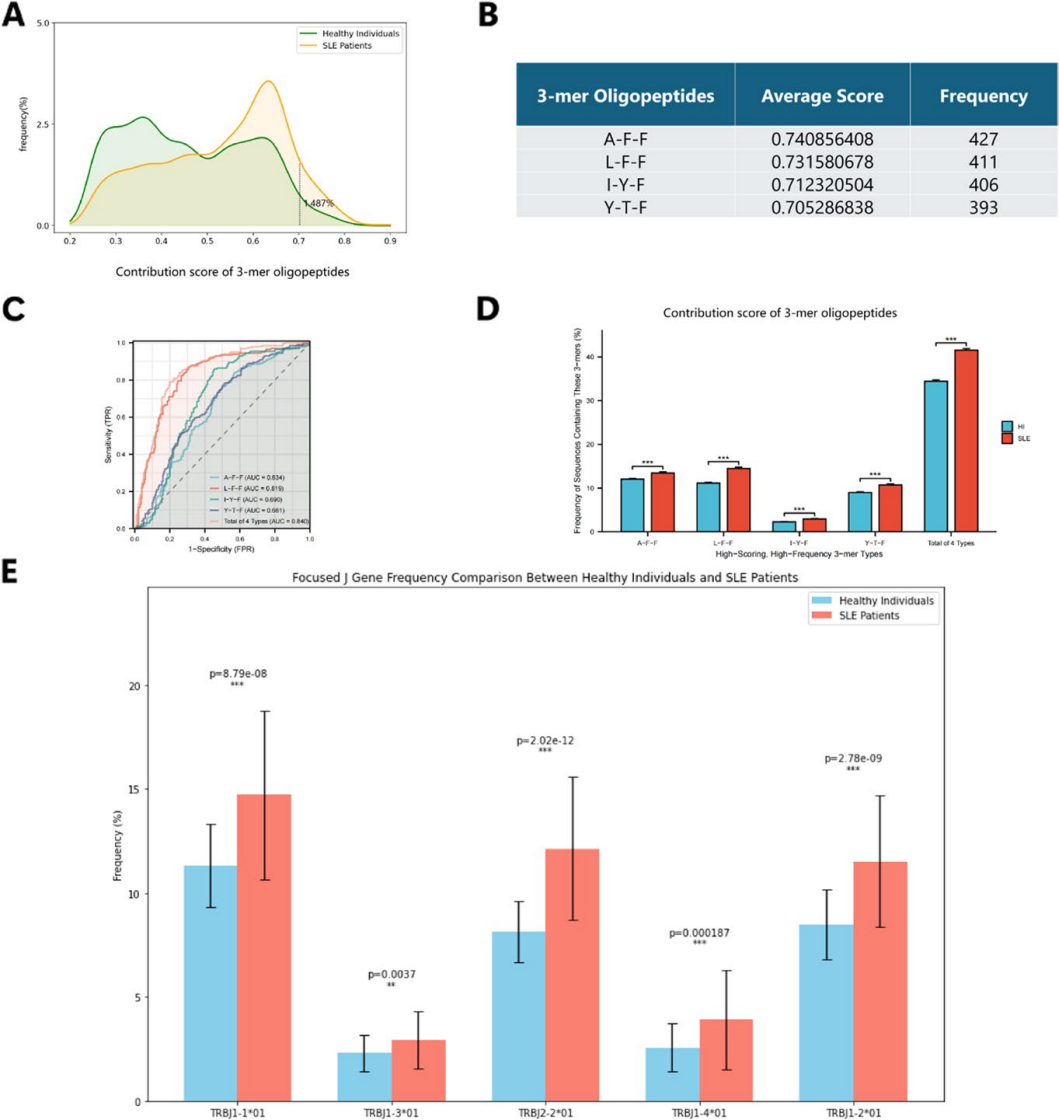
Screening of essential 3-mer oligopeptides and validating their repertoire classification performance. **A** Smoothed histogram reflecting the frequency distribution of 3-mer oligopeptide scores in SLE patients, where only a small fraction, less than 1.5%, achieves a high score of 0.7 or above, overlapping with that of healthy individuals. **B** Presentation of essential 3-mer oligopeptides after screening and their characteristics in the deep learning model. **C** ROC curve demonstrating good performance in diagnosing and classifying HI and SLE patients based on the frequency of essential 3-mer oligopeptides. **D** Clustered bar chart showing significant frequency differences of essential 3-mer oligopeptides in TCR samples from SLE patients compared to healthy individuals (HI) (independent samples t-test, $p < 0.001$); *$p < 0.05$, **$p < 0.01$, and ***$p < 0.001$. **E** Grouped bar chart showing significant differences in the frequency of J genes encoding essential 3-mers between healthy individuals and SLE patients

In the third fold of the DeepTAPE test set, the highest-scoring 2,000 sequences from each SLE patient sample were selected. In this process, each 3-mer oligopeptide was masked and subsequently input to the model for saliency analysis (see Methods). This methodology yielded high-scoring (0.7) and high-frequency 3-mer oligopeptides (Fig. 4A).

As a result, a specific set of essential 3-mer oligopeptides emerged that met the designated criteria: AFF, LFF, IYF, and YTF (Fig. 4B). Notably, the proportion of sequences containing these 3-mer oligopeptides in SLE samples was significantly higher than in HIs (independent samples t-test, $p < 0.001$). Furthermore, the total frequency of sequences encompassing these 3-mer oligopeptides was also markedly elevated in SLE samples (independent samples t-test, $p < 0.001$, as shown in Fig. 4D). Importantly, these oligopeptides exhibited significant frequency disparities between SLE patients and healthy individuals (HIs), thereby influencing the overall sample score.

Regarding the discrimination capability of these 3-mer oligopeptides, classification based on the frequency of sequences containing these oligopeptides in SLE patients and HIs yielded an area under the curve (AUC) exceeding 0.63 for all 3-mer oligopeptides. Notably, the classification outcome for the LFF oligopeptide achieved a remarkable AUC of 0.819. Additionally, when the cumulative frequency of all 3-mer oligopeptides was considered, the AUC elevated to 0.840, thereby establishing it as a promising biomarker candidate (Fig. 4C).

To better understand the biological basis of these findings, we investigated the positional distribution of these four essential 3-mers within the CDR3$\beta$ sequences. We found that they consistently and predominantly localized to the C-terminal third of the sequence (the tail region) (shown in Table 1). Given that the C-terminal region of CDR3 is largely encoded by the J-gene segment, this finding strongly points to a biased usage of specific J-genes. Indeed, frequency analysis of the corresponding J-genes revealed a pronounced and highly significant overrepresentation in SLE patients compared to healthy individuals (p < 0.001, independent *t*-test) (shown in Fig. 4E). 

**Table 1:** Distribution of 3-mers across the three one-third CDR3 segments and their corresponding J gene(s)

3-mer	Front (%)	Middle (%)	Tail (%)	J gene mutation
AFF	0.25	0.07	99.68	TRBJ1-1*01
LFF	0.23	0.27	99.49	TRBJ1-3*01
IYF	0.96	0.22	98.82	TRBJ2-2*01, TRBJ1-4*01
YTF	0.24	0.07	99.69	TRBJ1-2*01

For structural context, we selected the highest-frequency sequence containing each of the four essential 3-mer oligopeptides and predicted their tertiary structures using AlphaFold, which are provided for further reference (Supplementary result 2.5).

In addition to the globally identified motifs, we performed a focused analysis on the central region of the CDR3, adapting the method of Zhang et al. [56] by excluding the conserved ends. Although this approach identified other high-frequency 3-mers, a subsequent saliency analysis showed their contribution to SLE-associated CDR3 identification was relatively lower, indicating they are not suitable enough as potential biomarkers (Supplementary result 2.6).

### The role of model-identified gapped mer oligopeptides in enhancing diagnostic capabilities

**Fig. 5:**
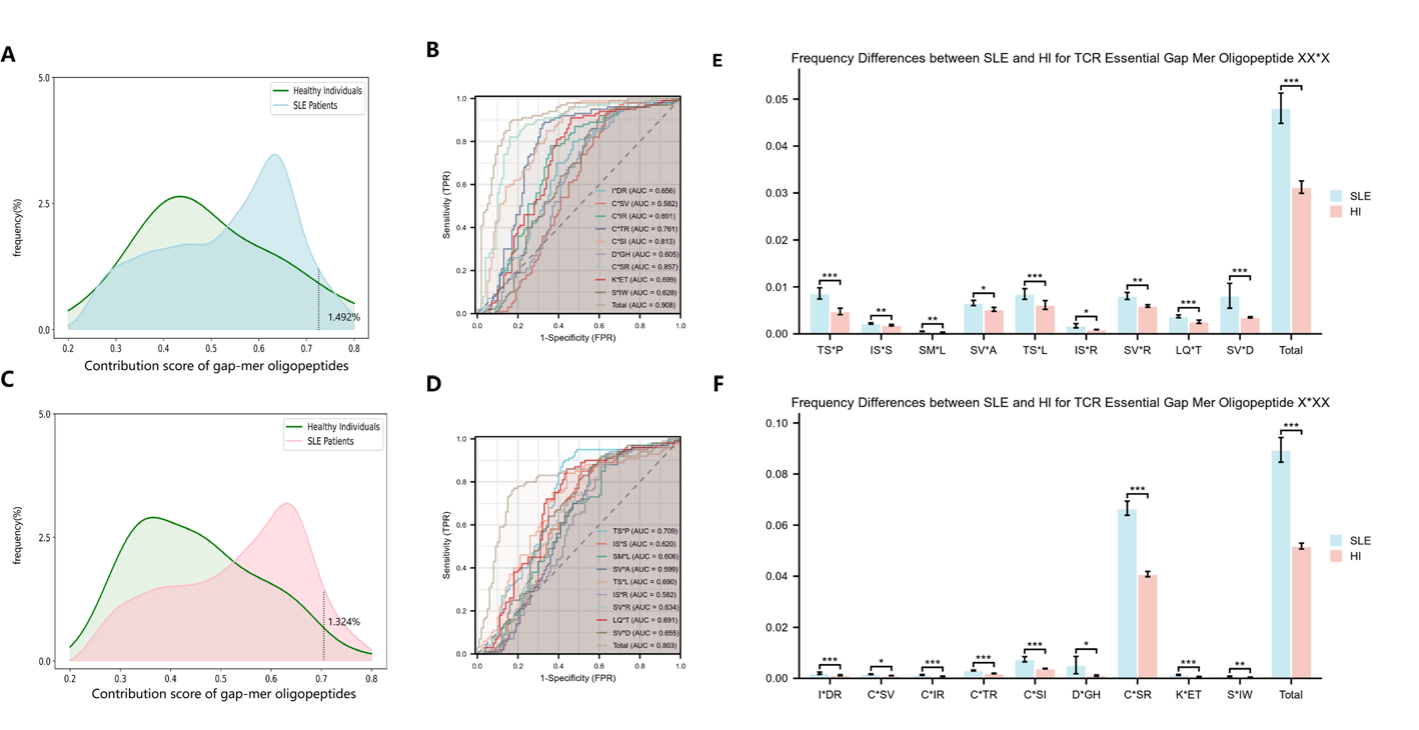
Screening of essential gapped mer oligopeptides and validating their repertoire classification performance. **A** The smoothed line histogram reflects the score frequency distribution of $X*XX$, with the threshold for the top 1.5% of scores set at 0.72, overlapping with that of healthy individuals. **B** The ROC curve illustrates the ability of several potential essential gapped mer oligopeptides $X*XX$ and their cumulative frequency to distinguish between SLE and healthy individuals (HI), along with their corresponding AUC. **C** The smoothed line histogram shows the score frequency distribution of $XX*X$, with the threshold for the top 1.5% of scores set at 0.70, overlapping with that of healthy individuals. **D** The ROC curve demonstrates the discriminative capability of several potential essential gapped mer oligopeptides $XX*X$ and their cumulative frequency for distinguishing SLE from HI, including their AUC. **E** The bar graph compares the frequencies of potential essential gapped mer oligopeptides $X*XX$ and their cumulative total, highlighting significant differences between SLE and HI. **F** The bar graph compares the frequencies of potential essential gapped mer oligopeptides $XX*X$ and their cumulative total, also revealing significant differences between SLE and HI. *$p < 0.05$, **$p < 0.01$, and ***$p < 0.001$

Through the application of masking techniques on high-scoring sequences, we further identified nine potential essential gapped mer oligopeptides of the form $X*XX$ (I*DR, C*SV, C*IR, C*TR, C*SI, D*GH, C*SR, K*ET and S*IW), and several of the form $XX*X$ (TS*P, IS*S, SM*L, SV*A, TS*L, IS*R, SV*R, LQ*T and SV*D), all scoring within the top 1.5% (Table 2 and Fig. 5A, C). For the $X*XX$ oligopeptides, we utilized the frequencies of these potential essential gapped mer oligopeptides and their total frequency to differentiate between SLE and healthy individuals (HI). This analysis revealed their potential as biomarkers, with the AUC values exceeding 0.55. Notably, C*SI and C*SR demonstrated exceptional reliability, achieving AUC values of 0.813 and 0.857, respectively. The cumulative frequency of all potential biomarkers reached an AUC of 0.908, approaching the discriminative capability of the deep learning model DeepTAPE, highlighting its potential as a powerful feature for disease classification and mechanistic investigation (Table 3 and Fig. 5B).

**Table 2:** Potential essential gapped 3-mer oligopeptides scores

Gapped mer	Score
X*XX	
I*DR	0.7318
C*SV	0.7642
C*IR	0.7287
C*TR	0.7358
C*SI	0.7431
D*GH	0.7300
C*SR	0.7305
K*ET	0.7276
S*IW	0.7246
XX*X	
TS*P	0.7200
IS*S	0.7099
SM*L	0.7201
SV*A	0.7172
TS*L	0.7170
IS*R	0.7076
SV*R	0.7320
LQ*T	0.7199
SV*D	0.7212

**Table 3:** Indices or biomarkers for the diagnosis of SLE and their performance

Diagnostic index or biomarker	AUC
**DeepTAPE**	
ARS [39]	0.979
**Essential 3-mer Oligopeptides**	
LFF	0.819
Total	0.840
Essential Gapped-mer X*XX	
C*SR	0.857
Total	0.908
Essential Gapped-mer XX*X	
TS*P	0.709
Total	0.803
**Other Indices or Biomarkers**	
SII [42]	0.678
UL95 [57]	0.703

Select only the best AUC for each index, and for the essential oligopeptides, include only the single one with the highest frequency of classification performance and the AUC results for the total frequency

Group comparisons of each essential gapped mer oligopeptide between SLE and HI revealed statistically significant differences ($p<0.05$), with most gapped mer oligopeptides exhibiting highly significant disparities (independent samples t-test, $p < 0.001$) (Fig. 5E).

In contrast, the $XX*X$ oligopeptides generally received slightly lower scores, indicating a weaker contribution to diagnostic capability compared to $X*XX$. AUC values remained above 0.55, but individual scores were predominantly in the range of 0.60 to 0.70. The cumulative frequency exhibited better biomarker potential, achieving an AUC of 0.804, which represents a viable biomarker candidate (Fig. 5D). Furthermore, group comparisons also demonstrated significant differences (independent samples t-test, $p<0.05$), with the aggregated frequency comparisons revealing substantial differences (independent samples t-test, $p < 0.001$) (Fig. 5F).

In this study, it is essential to conduct a comparative analysis of various diagnostic methods for systemic lupus erythematosus (SLE), including the machine learning-based DeepTAPE model, which has demonstrated specific advantages in several aspects. Furthermore, the essential 3-mer oligopeptides and gapped mer oligopeptides identified as potential biomarkers through the DeepTAPE approach offer additional insights. These biomarkers are evaluated against other diagnostic indices for SLE, such as the SII, despite the fact that these studies are based on different datasets and tasks.

### Potential antigens and genes for SLE identified from significant sequences by deep learning

We selected the highest-scoring 2,000 SLE-associated TCR CDR3 sequences from the third fold of the DeepTAPE test set, curating a set with a high likelihood of being implicated in SLE pathology. We hypothesize that these TCRs recognize specific SLE-associated autoantigens, driving the aberrant immune responses against self-tissues that are fundamental to SLE pathology.

Subsequently, a qualitative analysis was conducted using TCRanno to identify corresponding epitopes that align with the selected TCR clonotypes[53]. Following this, we performed a comprehensive query via the GeneCards database[55] to further verify the identified antigens and their related genes. Through rigorous examination of the pertinent academic literature, we refined our selection to include only those antigens that are relevant to autoimmune disorders (Table 4). These antigens emerge as promising candidates for future clinical and experimental validation as potential therapeutic targets in SLE [50, 58–65]. For example, CD109 has been found to have significant effects on rheumatoid arthritis; silencing CD109 or anti-CD109 treatment reduced the production of pro-inflammatory factors, cell migration, invasion, chemotactic attraction, and osteoclast differentiation, thereby decreasing the harmful inflammatory response of rheumatoid fibroblast-like synoviocytes (FLS) in vitro [58]. Similarly, IGPR plays a role in type 1 diabetes, as specific CD8 T cells (such as NRP-V7 specific T cells) can recognize it. These cells play a crucial role in the pathogenesis of type 1 diabetes by attacking the pancreatic beta cells, leading to insufficient insulin secretion [63]. Furthermore, the pathogenesis of SLE can be further explored along these lines, based on the potential genes and antigens that have been provided.

**Table 4:** Potential antigens and genes for SLE identified from significant sequences by deep learning

Potential antigen	Potential gene	Disease
CD109 antigen	*CD109*	Rheumatoid Arthritis
Insulin	*INS*	Type 1 Diabetes
Alpha-N-acetylgalactosaminide alpha-2,6-sialyltransferase 3	*ST6GALNAC1*	Colitis
Protein NPAT	*NPAT*	Ataxia-Telangiectasia
Islet-specific glucose-6-phosphatase-related protein	*IGRP*	Type 1 diabetes

## Discussion

Previous studies have elucidated the TCR repertoire in patients afflicted by immune-related disorders, thereby emphasizing the diagnostic potential of TCRs [31, 66, 67]. Despite these promising results, the application of deep learning to capture CDR3 features for the diagnosis of SLE remains insufficiently explored.

To address this critical gap, our recent research has developed DeepTAPE, a deep learning model based on a CNN-LSTM framework that utilizes the amino acid sequences of the TCR$\beta$ [39]. Notably, this model also integrates additional features, such as V genes and gene families, which have been reported to exhibit biased usage in autoimmune diseases and can serve as informative features for classification[31, 33, 68, 69]. Furthermore, the model incorporates residual connections within the CNN module, thereby enhancing its adaptability. Its effectiveness has been demonstrated through cross-validation and extensive independent assessments of external datasets.

In this study, we aim to provide a deeper understanding of our DeepTAPE model and validate its clinical utility. Our research reveals the significant potential of CDR3 in assessing SLE activity levels. Specifically, the ARS generated by DeepTAPE shows a positive correlation with the SLE Disease Activity Index (SLEDAI), with significant differences in ARS observed between SLE patients in active and silent states. This suggests its utility in classifying and determining patients’ disease activity status. Moreover, the ARS can essentially simulate the effectiveness of SLEDAI. By obtaining data through a simple examination of TCR sequencing from a patient’s PBMC, the DeepTAPE deep learning model can provide a more accessible, cost-effective, objective, and rapid complementary tool to SLEDAI, which relies on multiple tests and subjective assessments by physicians. From a medical and clinical perspective, this finding implies that differences among autoimmune patients with varying activity levels are reflected in the TCR$\beta$ distribution. This observation can further serve as an immunologically based tool to assist clinicians in evaluating the activity status of a patient’s condition.

Furthermore, building on DeepTAPE’s application in assessing SLE activity, our analysis has identified specific biomarkers that deepen our understanding of the immune mechanisms underlying SLE. Our focus on short oligopeptides is guided by established findings in crystallography studies and TCR recognition. Structural analyses have revealed that key amino acid patterns within the CDR3 loop help define the functional core of TCR-peptide interactions [48]. The importance of such motifs in autoimmunity was highlighted by Chamoto et al., who demonstrated their role in regulating autoreactivity [70]. In the context of SLE, where genetic predisposition is linked to specific HLA alleles that function as risk factors [71], the peptide-presenting role of these molecules suggests that SLE-associated TCRs may share common motifs for recognizing disease-related pMHC complexes. This hypothesis forms the basis of our approach to identify these oligopeptides as potential functional units and biomarkers. To achieve this, we employed a mask-based salience analysis on high-scoring sequences, a focused approach that identifies the most salient molecular features driving classification. This analysis pinpointed four critical 3-mer oligopeptides (AFF, LFF, IYF, YTF) and nine key gapped-mer oligopeptides.

These motifs, found to be significantly elevated in frequency among SLE patients, are thus positioned as potential biomarkers for dissecting disease-specific immune responses. Notably, the gapped-mer oligopeptides demonstrated superior discriminative performance. We attribute this success to their ability to represent interactions between non-contiguous residues–a complex feature our model is uniquely suited to capture. This capability stems directly from its hybrid architecture: the CNN component excels at identifying local motifs and conserved structural features [72], while the LSTM is adept at modeling the long-range, contextual dependencies that connect these non-contiguous residues [73]. Residual connections further stabilize this deep architecture, ensuring it learns these intricate patterns effectively while mitigating overfitting. This synergistic design is therefore crucial for generalizing the functional patterns on CDR3 sequences. Nonetheless, these preliminarily identified oligopeptides require further validation. Subsequent steps should include *in silico* analysis, such as protein structural docking and public database cross-validation, as well as crucial in vitro functional assays to confirm their biological role and investigate the underlying TCR-epitope binding mechanisms in SLE.

Despite the demonstrated effectiveness of DeepTAPE and its advantages for clinical and disease mechanisms research, several challenges and opportunities for improvement remain. Firstly, while DeepTAPE can identify repertoires with various autoimmune diseases from healthy cohorts, it lacks the specificity to distinguish among different autoimmune conditions. This limitation highlights the need for a broader and more diverse dataset of TCRs to train a multi-classification deep learning model capable of diagnosing and distinguishing these diseases. Secondly, our sequence-based motif analysis is limited as it does not incorporate the structural context of pMHC interaction. Therefore, elucidating how these identified motifs contribute to binding the pMHC complex requires further investigation through molecular modeling and wet-lab validation. In addition, our focus on short 3-mers and gapped-mers may only capture fragments of the complete binding interface. Future computational work should also integrate these biophysical features and explore more complex motifs to better model the underlying interactions. Furthermore, our model currently uses V gene categories; a more granular approach could be explored by incorporating the full TCR sequence, integrating the V and J gene sequences with the CDR3. This represents a valuable direction for enhancing performance, where the inherent challenge of accurately extracting information from such complex sequences could be addressed by employing advanced TCR encoders and structure-based feature extraction methods, such as TCR2vec [74] and TCRen [75, 76]. As our salience analysis reveals position-specific contributions of motifs, future architectural designs could be enhanced by incorporating this spatial context, for instance, through positional encoding or attention layers. Moreover, the ARS has indeed shown, based on our current computational research and analysis, to effectively reflect the disease activity levels in SLE patients; however, further clinical validation is necessary. Additionally, its capability to assess the extent of damage caused by the disease requires enhancement. Future studies should consider incorporating more sophisticated damage assessment methodologies, such as standardized clinical damage indices (e.g., SLICC/ACR DI) or organ-specific damage scores, which may provide more clinically relevant correlations with our TCR-based biomarkers. Furthermore, longitudinal studies tracking damage progression over time would be valuable for validating the predictive utility of ARS for long-term outcomes. The biomarkers we identified, as well as potential SLE-associated genes and antigens, also necessitate further medical validation and clinical research to pave the way for a deeper exploration of SLE in the future.

In summary, DeepTAPE is a pioneering deep-learning model that assists in SLE diagnosis through the CDR3 sequence features, offering insights into potential antigen identification and setting the stage for further advancements in immunodiagnostics.

## Conclusion

In summary, the DeepTAPE framework offers a novel perspective for investigating autoimmune disorders, particularly SLE. The robust performance in the SLE classification task, along with the linear association between the ARS and clinical evaluations, underscores the promising potential of DeepTAPE in supporting diagnostic assessments while providing a quantitative measure of disease severity from immune repertoire data. Furthermore, the insights gained from utilizing DeepTAPE may inform the development of novel biomarkers that facilitate earlier and more accurate diagnoses of various autoimmune disorders.

Furthermore, the identification of specific oligopeptides, such as the critical 3-mer and gapped-mer sequences, not only enhances our current understanding but also opens avenues for future research into their roles as potential biomarkers in other related conditions. This work underscores the importance of leveraging advanced deep learning techniques to explore the complexities of TCR sequences, thereby significantly enhancing our understanding of immune system dynamics. The continued validation of these biomarkers is a critical step toward establishing DeepTAPE as a transformative tool in autoimmune disease management, ultimately leading to more personalized and effective treatment strategies.

## Supplementary Information

Supplementary Material 1

## Data Availability

The datasets employed in this study are publicly available. The data used for both model training and testing were obtained from previously published works, with all ethical standards rigorously upheld during the original collections. The main training dataset for SLE patients and healthy cohorts is derived from the study by Liu et al. [31]. The external independent test dataset for Juvenile Idiopathic Arthritis (JIA) is sourced from Henderson et al. [44] (available at https://clients.adaptivebiotech.com/pub/Henderson-2015). The external independent test dataset for Autoimmune Arthritis (AutoA) is from Bonami et al. [45] (available at https://clients.adaptivebiotech.com/pub/bonami-2021-biorxiv), and the negative control set consists of a random mixture of three datasets provided by Emerson et al. [77] (available at https://clients.adaptivebiotech.com/pub/emerson-2017-natgen), Lee et al. [78] (https://clients.adaptivebiotech.com/pub/lee-2022-jcp), and Mitchell et al. [79] (https://clients.adaptivebiotech.com/pub/mitchell-2022-jcii).

## Abbreviations

SLE: Systemic Lupus Erythematosus
TCR: T Cell Receptor
CDR3: The Third Complementarity-Determining Region
DeepTAPE: Deep Learning-based TCR$\beta$-utilized Autoimmune Disease Prediction Engine
Bi-LSTM: Bidirectional Long Short-Term Memory
CNN: Convolutional Neural Network
HIs: Healthy Individuals
JIA: Juvenile Idiopathic Arthritis
AutoA: Autoimmune Arthritis
PBMC: Peripheral Blood Mononuclear Cells
ARS: Autoimmune Risk Score
SLEDAI: Systemic Lupus Erythematosus Disease Activity Index
PCC: Pearson Correlation Coefficient
CR: Correlation Ratio

## Code availability

The code used for this study is publicly available at the following GitHub repository: https://github.com/deepomicslab/DeepTAPE-1.0. This repository contains the detailed documentation and scripts to reproduce the results presented in this manuscript.
