# Supplementary material for: Deep learning-driven TCR[image] repertoire analysis enhances diagnosis and enables mining of immunological biomarkers in systemic lupus erythematosus
Source: BioData Min. 2025 Oct 31;18:76. doi: 10.1186/s13040-025-00490-5 (PMC12577242; doi:10.1186/s13040-025-00490-5)
Supplement: Supplementary file 1 — Supplementary Material 1 [file 13040_2025_490_MOESM1_ESM.pdf]

Supplementary: Deep Learning-Driven TCRβ Repertoire Analysis Enhances Diagnosis and Enables Mining of Immunological Biomarkers in Systemic Lupus Erythematosus

Tongfei Shen[1†], Yifei Sheng[1†], Wan Nie[1], Shuo Yang[1], Kaiqi Li[1], Ziwei Ma[1], Zhao Ling[1], Bowen Tan[1], Xikang Feng[2*], Miaozhe Huo[1*]

*Corresponding author(s). E-mail(s): fxk@nwpu.edu.cn; miaozhhuo2-c@my.cityu.edu.hk;
Contributing authors: timshen2-c@my.cityu.edu.hk; yifesheng2-c@my.cityu.edu.hk; wannie2-c@my.cityu.edu.hk; syang58-c@my.cityu.edu.hk; kaiqili2-c@my.cityu.edu.hk; ziweima3-c@my.cityu.edu.hk; zhaoling2-c@my.cityu.edu.hk; bowentan3-c@my.cityu.edu.hk;
†These authors contributed equally to this work.

1 Supplemental Methods

1.1 Public immune repertoire dataset information

The main dataset utilized in this investigation was procured from publicly accessible databases and comprises TCR sequences extracted from the Peripheral Blood Mononuclear Cells (PBMCs) of 439 healthy individuals (HIs) and 877 patients diagnosed with SLE[1]. For a comprehensive description of the library preparation, sequencing protocols, and initial data processing, please refer to the original publication. Demographic characteristics of the dataset are shown in Supplementary Table S5. This data was employed for both the training and cross-validation phases of our framework.

To rigorously assess the predictive efficacy of the model on independent samples, we included 37 samples from patients with Juvenile Idiopathic Arthritis (JIA) and 33 samples from individuals with Autoimmune Arthritis (AutoA). Although significant differences exist among SLE, JIA, and AutoA in terms of genetic susceptibility, target organ involvement patterns, and core pathogenic pathways[2, 3], aberrant clonal expansions and diversity imbalances in the TCR CDR3 region show some degree of overlap among JIA, AutoA, and SLE, which may indicate similar pathogenic origins or comparable autoimmune consequences underlying dysregulated adaptive immune responses[3–7]. To establish negative controls, we selected three distinct cohorts of healthy individuals, all sourced from public datasets[8–10]. The TCR sequences for the independent testing cohort and the healthy controls were also derived from PBMCs. This methodology ensures a comprehensive and robust evaluation of our model's performance across varied clinical contexts.

1.2 Repertoire data processing details

All the repertoires were split into training and validation sets at a ratio of 4:1. In the training dataset, we conducted a random selection to choose an equal number of individuals from both healthy donors and SLE patients. Within the TCR repertoire of each selected individual, each valid TCR CDR3 sequence underwent rigorous filtering based on the following criteria: (i) the sequence length must be between 10 and 24 amino acids; (ii) the sequence must consist only of standard amino acids; (iii) the sequence should start with cysteine (C) and end with phenylalanine (F); and (iv) the variable gene locus must be identifiable. Considering computational efficiency and the richness of information, we retained the 2,000 most frequent TCR clone types from each individual to represent their immunological profile.

Subsequently, repertoire-level labels were assigned to individual TCR sequences. Specifically, representative CDR3 sequences from healthy individuals were categorized as SLE-unrelated TCRs (negative samples), whereas only those sequences derived from SLE patients that were absent from the healthy repertoires were designated as SLE-related TCRs (positive samples). Following TCR label assignment, duplicate clonotypes were removed for each category. Then, negative samples were down-sampled to balance the number of positive and negative TCRs. Finally, the curated sequences were randomly shuffled and split in a 4:1 ratio to establish training and validation datasets.

To systematically evaluate the impact of different feature combinations on model performance, we designed datasets incorporating varying levels of genetic information beyond the CDR3 sequence alone. Guided by findings from our primary data source[1], which indicated that the J-gene offered limited additional discriminatory power for SLE repertoire classification, we focused our feature engineering on V-gene usage to build a more parsimonious and robust model. Following this rationale, we constructed two such datasets, named 'CDR3-V gene' and 'CDR3-V gene family.' These were created by integrating V gene or V gene family information alongside the CDR3 amino acid sequences. During the calculation of clonotype frequencies and the deduplication process of TCR data, we considered both the V gene type and the CDR3 sequences, rather than focusing solely on the CDR3. Consequently, we obtained a training dataset that consists of a sequence set $S = \{s_1, s_2, s_3, \ldots, s_n\}$, a gene set $G = \{g_1, g_2, g_3, \ldots, g_n\}$, and the corresponding label set $T = \{t_1, t_2, t_3, \ldots, t_n\}$ which contains the TCR-level labels. The sequence set S, gene set G, and label set T each comprise n elements, ensuring precise one-to-one correspondence among them.

Similarly, we construct independent datasets JIA and AutoA datasets.

1.3 Model architecture

We trained the TCR classifier model using three datasets with different feature combinations: the first uses only amino acid sequences, the second combines these with V gene features, and the third integrates V gene family features. The model integrates a convolutional neural network (CNN) combined with long short-term memory (LSTM) layers and residual connections.

The model accepts two types of inputs: CDR3 amino acid sequences and V gene features (optional). Each input type is tokenized using respective dictionaries. An embedding layer transforms these sequences into fixed-length vectors of 24 dimensions. To standardize sequence lengths, a zero-padding method is applied, padding shorter sequences at the beginning. For the V gene features, which are treated as categorical inputs (by mutation type or V gene family), a trainable embedding layer converts each category into a 20-dimensional vector. This dimensionality is a hyperparameter chosen to provide sufficient representational capacity for the V gene while controlling model complexity.

The CNN architecture comprises four convolutional blocks, each consisting of two convolutional layers followed by a max pooling layer. The convolutional layers use ReLU activation and are equipped with 256 filters each. The max pooling layers have a pool size of 5 and a stride of 1. The convolution operations are defined by:

$$c_{i,j} = \sum_{k=0}^{K-1} W_{k,j} \cdot s_{i+k} + b_j$$

where $W_{k,j}$ is the weight of the j-th filter, b_j is the bias, and K is the filter size. Residual connections allow each block's input to derive from both its predecessor and all prior blocks. Outputs from the convolutional layers are flattened and then passed to a fully connected layer with 16 neurons and Sigmoid activation.

The LSTM component includes three bidirectional layers with a dropout rate of 0.2 to mitigate overfitting.

Outputs from the CNN and LSTM feature extractors are concatenated, either with or without the V gene feature embedding, and then passed through a connected layer with Sigmoid activation for binary classification to fit the assigned TCR labels.

1.4 Model training and evaluation

The TCR classifiers were trained using the RMSprop optimizer, initiated with a learning rate of 0.0001. Binary cross-entropy was employed as the loss function, and the models achieved convergence after five epochs.

For model validation, we employed 5-fold cross-validation using 439 HI samples and 439 SLE patient samples, which were randomly selected from the total patient population. For each individual, the DeepTAPE classifier aggregated the results from the top 2,000 most frequent representative TCRs in an individual's repertoire to compute an autoimmune risk score (ARS), which indicates the probability of the individual having SLE. The ARS is calculated using the following expression:

$$\text{ARS} = \frac{\sum_{i=1}^{n} \text{TCR classifier}(\text{tcr}_i)}{n}. \tag{S1}$$

Here, tcr_i denotes the i-th TCR in the individual's set of n representative TCRs, where n equals 2,000. The TCR classifier represents a well-trained classifier at the TCR level, which assesses each TCR for its association with SLE.

The threshold for ARS was determined using the training dataset. In the validation phase, individuals with ARS values exceeding this threshold were classified as having SLE, while those below were considered healthy.

The model's performance metrics, including accuracy, precision, recall, F1-score, and the area under the curve (AUC), were calculated using standard formulas.

1.5 Baseline models

To establish the effectiveness of TCR classifier, we compared its performance against several benchmark classifiers, each also utilizing amino acid sequences and gene frequency information.

CNN-LSTM: Similar in structure and hyperparameters to DeepTAPE, but lacks residual connections.

CNN: Comprising four convolutional layers paired with max-pooling layers, this model utilizes a kernel size of 5.

Bi-LSTM: This model features three layers of bidirectional LSTM, each with a dropout rate of 0.2.

SimpleRNN: Composed of two layers of recurrent neural networks (RNN), with a dropout rate of 0.2.

The classification performance of DeepTAPE was also evaluated in comparison to the Random Forest (RF) classifier, as proposed in a previous study[1]. The RF classifier has demonstrated effectiveness in distinguishing between SLE patients and healthy individuals by analyzing variations in individual V gene frequencies. We implemented the RF model adhering to the parameters specified in the original publication to ensure a consistent and fair comparison.

1.6 Independent test on non-SLE autoimmune diseases

The model underwent independent testing using external datasets from other autoimmune diseases, including JIA and AutoA datasets. Negative samples used for independent testing came from the external HI repertoires[10–12]. Considering the potential differences in the V gene and V gene family distributions of TCRs from other diseases compared to SLE, a self-adaptive mechanism based on the Pearson Correlation Coefficient (PCC) was introduced. This mechanism determines whether to utilize a specific gene feature based on the ratio of the gene frequency distribution correlation coefficient between the target disease and SLE samples versus the correlation coefficient between HI samples. The standard frequency distributions are derived from the average frequency distributions of 10 samples each from HI and SLE patients. For a sample's gene frequency distribution f_{SA} and a standard frequency distribution f_{ST}, the Pearson correlation coefficient formula is given by:

$$\mathrm{cor}(f_{\mathrm{SA}}, f_{\mathrm{ST}}) = \frac{\sum_{i=1}^{n}(f_{\mathrm{SA},i} - \bar{f}_{\mathrm{SA}})(f_{\mathrm{ST},i} - \bar{f}_{\mathrm{ST}})}{\sqrt{\sum_{i=1}^{n}(f_{\mathrm{SA},i} - \bar{f}_{\mathrm{SA}})^2}\sqrt{\sum_{i=1}^{n}(f_{\mathrm{ST},i} - \bar{f}_{\mathrm{ST}})^2}} \tag{S2}$$

The Correlation Ratio (CR) is defined as:

$$\mathrm{CR} = \frac{\mathrm{cor}(f_{\mathrm{SA}}, f_{\mathrm{SLE}})}{\mathrm{cor}(f_{\mathrm{SA}}, f_{\mathrm{HI}})} \tag{S3}$$

1.7 Statistical analysis of ARS for SLE disease activity

To evaluate whether the difference in the ARS between healthy individuals and SLE patients, as well as between active and silent states among patients, is statistically significant, a two-sample t-test was performed. The test statistic is calculated as follows:

$$t = \sqrt{\frac{n_1 + n_2}{n_1 n_2}} \cdot \frac{\bar{X}_1 - \bar{X}_2}{\sqrt{\frac{(n_1-1)s_1^2+(n_2-1)s_2^2}{n_1+n_2-2}}}$$

and the corresponding p-value is computed by

$$p = 2 \cdot P(T \geq |t|)$$

Pearson's correlation coefficient and Spearman's rank correlation coefficient were employed to assess relationships between ARS and clinical indicators; formulas and testing procedures are as described in the manuscript.

2 Supplemental Results

2.1 TCR repertoire classification performance in SLE using sequence and gene features

A previous study demonstrated the potential of TCR repertoire gene information for SLE detection, though its clinical utility requires cautious interpretation considering

genetic heterogeneity[1]. This was achieved through a Random Forest (RF) algorithm that effectively distinguished between SLE patients and healthy individuals based on V gene frequencies, yielding promising outcomes. Moreover, several effective models have been developed for diagnosing various diseases by leveraging the amino acid sequences of the CDR3 in TCR's β chain. In light of these findings, we adopted a neural network approach to identify autoimmune diseases, including SLE, from TCR repertoire information by integrating CDR3 sequences and gene usage features.

When compared to other classifiers that utilize TCR β CDR3 amino acid sequences and gene frequency information, the DeepTAPE model, built on a CNN-LSTM architecture with residual connections, demonstrated superior performance across five metrics. The minimal performance variance observed during the 5-fold cross-validation indicates the model's stability (Tab. S1). According to the confusion matrix, all three DeepTAPE input modes, which utilize different feature combinations, exhibited commendable classification performance. Notably, the DeepTAPE input mode that additionally incorporated V gene and V gene family features displayed a lower misclassification rate compared to the model utilizing only amino acid sequences, indicating improved classification AUC. Most misclassifications across the three models were categorized as false negatives (i.e., patient samples incorrectly identified as healthy controls) (Fig. S1F, G, H).

2.2 Assessing the cross-disease generalizability of DeepTAPE in autoimmune disorders

The three input versions of the DeepTAPE model were initially trained using data derived from SLE patients and HIs. The performance of these models varies markedly depending on the feature combinations implemented. While they exhibit strong classification capabilities for SLE, they also demonstrate some level of discrimination proficiency for other autoimmune disorders. In this context, samples from juvenile idiopathic arthritis (JIA) and autoimmune arthritis (AutoA), alongside samples from healthy individuals (HIs), were utilized to establish an external independent test set.

Equal quantities of patient samples and HI samples were randomly selected and input into the model for prediction, with this procedure repeated five times to derive average results. The findings revealed that for JIA, the AUC of the DeepTAPE model employing exclusively amino acid sequences achieved a value of 86.98%. However, when the gene usage feature was included, the average AUC for the DeepTAPE model that integrated amino acid sequences with V genes and those combined with V gene families decreased to 64.22% and 63.33%, respectively (shown in Fig. S5A). A similar trend was noted for AutoA, where the average AUC utilizing amino acid sequences was higher at 85.78%, compared to average AUCs of 74.89% and 77.62% for the models that incorporated amino acid sequences with V genes and V gene families, respectively (Fig. S5B and Tab. S2). Moreover, the confusion matrix results indicated that the discrimination performance of the DeepTAPE model based solely on amino acid sequences was significantly superior for both JIA and AutoA (Fig. S5C, D).

The ability of the DeepTAPE model, using only CDR3 amino acid sequences, to effectively distinguish patients with JIA and AutoA from HIs demonstrates its cross-disease generalizability. This finding suggests that the CDR3 features learned from the

SLE dataset might have captured underlying patterns of immune dysregulation that are common across these distinct autoimmune disorders[13]. Such trans-disease signatures are biologically plausible, with known examples like PTPN22 variants acting as a shared genetic risk factor for multiple rheumatic diseases [14] and a common type I interferon pathway being activated across SLE, myositis, and rheumatoid arthritis [15]. Conversely, the sharp decline in generalization performance upon the inclusion of V gene usage features implies that, unlike the CDR3 sequence motifs, V gene features are highly disease-specific. This divergence may be driven by distinct, disease-specific mechanisms that shape the V gene frequency distribution differently in each condition. Our analytical assessments support this conclusion, revealing significant discrepancies in the V gene and V gene family distributions between the TCR repertoires of JIA/AutoA patients and those of the SLE patients used for training (Fig. S5E, F). These disparate frequency profiles likely introduced confounding signals, thereby degrading DeepTAPE's classification performance for non-SLE diseases.

To resolve this challenge, the DeepTAPE model incorporated a self-adaptive mechanism based on the Pearson correlation coefficient (PCC), which facilitates a comparison between the frequency distributions of V genes or V gene families in the independent test set and those of SLE patients and HIs. The results indicated that the correlation coefficients for the V gene or V gene family distributions between JIA patients and HIs were significantly greater than those between JIA patients and SLE patients ($p < 0.001$). A similar observation is noted for AutoA, which complicates effective classification (Fig. S5G, H). The self-adaptive mechanism utilizing PCC enables the model to autonomously select the most appropriate feature combinations tailored to each specific disease. Thus, this mechanism is essential for the automatic determination of classification feature compositions for each condition, as evidenced by the model's choice to rely solely on amino acid sequences for classifying JIA and AutoA.

2.3 Comparison of V- and J-gene usage in TCR clonotype with and without essential 3-mer oligopeptides

The association between the presence of the essential 3-mer oligopeptides LFF, AFF, YTF and IYF and biased TRBV/TRBJ usage was investigated. From a cohort of systemic lupus erythematosus (SLE) patients, CDR3β sequences containing at least one of the four essential 3-mers were identified and compared to CDR3β sequences that did not contain these motifs.

Allele-level analysis of TRBJ usage revealed significant biases. Several TRBJ alleles, including TRBJ2-7*01, TRBJ1-1*01 and TRBJ1-2*01, were found to be significantly over- or under-represented among sequences containing essential 3-mers relative to motif-negative sequences (two-sided test, adjusted $p < 0.001$) (Fig. S3A).

Analysis of TRBV usage indicated an even broader spectrum of biased V genes. Multiple TRBV alleles, notably TRBV11-2*01, TRBV11-2*03 and TRBV20-1*01, were significantly enriched among sequences encoding LFF, AFF, YTF or IYF (two-sided test, adjusted $p < 0.001$) (Fig. S3B).

These observations support the biological relevance of the essential 3-mer oligopeptides LFF, AFF, YTF and IYF in SLE. The presence of these motifs is associated with

selective TRBV and TRBJ usage and clonal enrichment in the CDR3β repertoire of affected individuals.

2.4 Comparison of V- and J-gene usage in TCR clonotype with and without essential 3-mer oligopeptides

The association between presence of the essential 3-mer oligopeptides LFF, AFF, YTF and IYF and biased TRBV/TRBJ usage was investigated. From a cohort of systemic lupus erythematosus (SLE) patients, CDR3β sequences containing at least one of the four essential 3-mers were identified and compared to CDR3β sequences that did not contain these motifs.

Allele-level analysis of TRBJ usage revealed significant biases. Several TRBJ alleles, including TRBJ2-7*01, TRBJ1-1*01 and TRBJ1-2*01, were found to be significantly over- or under-represented among sequences containing essential 3-mers relative to motif-negative sequences (two-sided test, adjusted $p < 0.001$) (Fig. S3A).

Analysis of TRBV usage indicated an even broader spectrum of biased V genes. Multiple TRBV alleles, notably TRBV11-2*01, TRBV11-2*03 and TRBV20-1*01, were significantly enriched among sequences encoding LFF, AFF, YTF or IYF (two-sided test, adjusted $p < 0.001$) (Fig. S3B).

These observations support the biological relevance of the essential 3-mer oligopeptides LFF, AFF, YTF and IYF in SLE. The presence of these motifs is associated with selective TRBV and TRBJ usage and clonal enrichment in the CDR3β repertoire of affected individuals.

2.5 Structures of high-frequency sequences containing essential 3-mer oligopeptides

Through targeted screening, we identified the most frequent sequences containing essential 3-mer oligopeptides within the entire SLE patient dataset. These sequences include: CASSQDSVSEAFF, CAWSLRGGTGELFF, CASSQVVSSGNTIYF, and CASSLDSDNYGYTF. We subsequently predicted their tertiary structures using AlphaFold. The resulting structural models are shown in Fig. S4. Additional structural data and models have been made available on our GitHub repository.

2.6 Identification of potential 3-mers from the CDR3 central region

Adopting the preprocessing method from the GINNA project[16], we focused our analysis on the most variable segment of the CDR3 sequences. Specifically, we truncated the first three and the last two amino acids of each sequence, defining the remaining portion as the central region. Using a screening threshold where a k-mer had to appear in over one-tenth of the SLE-associated TCR clonotypes, we identified three high-frequency oligopeptides from the central regions: GEQ, ETQ, and TGE.

We then analyzed the positional distribution of these central region 3-mers within the CDR3 sequences of SLE patients. Unlike the globally identified essential 3-mers

(LFF, AFF, YTF or IYF), which are often tied to the J-gene region, these oligopeptides are predominantly located in the middle of the CDR3β sequences (Supplemental Table S3). Furthermore, when we employed saliency analysis to quantify their potential contribution, the results showed that their scores for identifying SLE-associated CDR3s were substantially lower than those of the four globally identified 3-mers (Supplemental Table S4), suggesting a limited role in disease association.

3 Supplemental Tables

Table S1: Comparison of the SLE classification performance using 5-fold cross-validation

Model	AUC	Accuracy	Precision	Recall	F1-score
			**Only V Gene Frequency**		
VGene-RF[1]	96.11% ± 1.70%	88.97% ± 3.38%	91.23% ± 4.49%	86.41% ± 4.26%	88.68% ± 3.47%
			**CDR3 Amino Acid Sequence**		
CNN-LSTM	97.46% ± 0.69%	92.82% ± 1.53%	91.21% ± 2.68%	93.87% ± 1.81%	**92.97% ± 1.43%**
CNN	95.65% ± 2.10%	90.39% ± 3.01%	90.71% ± 4.78%	90.26% ± 4.85%	90.37% ± 3.02%
Bi-LSTM	97.30% ± 0.80%	92.69% ± 1.40%	91.44% ± 3.77%	91.03% ± 5.59%	92.53% ± 1.67%
SimpleRNN	96.25% ± 1.64%	91.41% ± 1.73%	90.07% ± 3.63%	93.33% ± 4.29%	91.56% ± 1.74%
**DeepTAPE**	**97.52% ± 0.68%**	**92.82% ± 1.39%**	**91.84% ± 4.74%**	**94.36% ± 3.46%**	92.95% ± 1.21%
			**CDR3 Amino Acid Sequence + V Gene**		
CNN-LSTM	97.43% ± 0.83%	92.95% ± 4.33%	92.30% ± 3.26%	93.85% ± 2.07%	93.02% ± 3.45%
CNN	94.36% ± 5.94%	88.85% ± 2.74%	88.86% ± 5.08%	89.49% ± 7.77%	88.86% ± 2.98%
Bi-LSTM	96.85% ± 2.43%	92.05% ± 9.02%	91.43% ± 2.93%	92.05% ± 3.92%	92.05% ± 9.40%
SimpleRNN	97.67% ± 0.89%	93.33% ± 2.06%	92.01% ± 4.09%	**95.28% ± 9.02%**	93.48% ± 8.45%
**DeepTAPE**	**97.70% ± 0.86%**	**93.56% ± 1.80%**	**92.38% ± 3.69%**	95.15% ± 1.78%	**93.69% ± 1.57%**
			**CDR3 Amino Acid Sequence + V Gene Family**		
CNN-LSTM	97.85% ± 0.76%	93.46% ± 1.05%	92.82% ± 2.91%	94.36% ± 2.95%	93.52% ± 1.04%
CNN	94.16% ± 1.71%	88.46% ± 2.48%	89.32% ± 4.17%	87.69% ± 5.26%	88.35% ± 2.58%
Bi-LSTM	97.37% ± 1.19%	93.33% ± 2.01%	92.80% ± 3.48%	94.10% ± 2.14%	93.40% ± 1.87%
SimpleRNN	97.26% ± 0.83%	92.95% ± 1.63%	92.08% ± 3.13%	94.10% ± 1.46%	93.05% ± 1.52%
**DeepTAPE**	**97.99% ± 0.82%**	**93.97% ± 1.61%**	**93.70% ± 2.57%**	**94.36% ± 2.14%**	**94.00% ± 1.57%**

The table outlines the effectiveness of various predictive models in SLE and HI repertoire classification on the validation dataset. Bold values represent the best performance, while underlined values indicate the second-best performance within each metric across methods.

Table S2: Performance of the DeepTAPE model across different input modes on non-SLE autoimmune disease datasets.

Input modes	AUC	Accuracy	Precision	Recall	F1-score
			**AutoA**		
DeepTAPE (AA Seqs + V Gene Families)	77.62% ± 1.25%	80.33% ± 0.67%	**93.44% ± 1.91%**	65.33% ± 2.67%	76.83% ± 1.26%
DeepTAPE (AA Seqs + V Genes)	74.89% ± 0.78%	76.00% ± 0.82%	90.86% ± 3.21%	58.00% ± 2.67%	70.70% ± 1.43%
**DeepTAPE (Only AA Seqs)**	**95.78% ± 0.19%**	**90.33% ± 1.94%**	92.18% ± 4.83%	**88.67% ± 5.42%**	**90.14% ± 2.04%**
			**JIA**		
DeepTAPE (AA Seqs + V Gene Families)	63.33% ± 1.84%	62.00% ± 2.45%	71.47% ± 8.13%	44.67% ± 15.00%	52.50% ± 9.65%
DeepTAPE (AA Seqs + V Genes)	64.22% ± 1.26%	71.00% ± 1.33%	**86.40% ± 4.14%**	50.00% ± 0.00%	63.31% ± 1.08%
**DeepTAPE (Only AA Seqs)**	**86.98% ± 1.47%**	**82.67% ± 3.09%**	82.67% ± 2.08%	**82.67% ± 6.46%**	**82.55% ± 3.81%**

Bold values represent the best performance, while underlined values indicate the second-best performance within each metric across three encoding modes. AA seq, amino acid sequence.

Table S3: Positional distribution (%) of selected 3-mers within SLE cohort CDR3β sequences

3-mer	Front (%)	Middle (%)	Tail (%)
GEQ	1.01	90.03	8.96
ETQ	8.03	71.92	20.05
TGE	0.20	92.35	7.46

Table S4: Average score and frequency of 3-mers in the CDR3 central region

3-mer	Average Score	Frequency
GEQ	0.1541	335
ETQ	0.1033	246
TGE	0.0953	346

4 Supplemental Figures

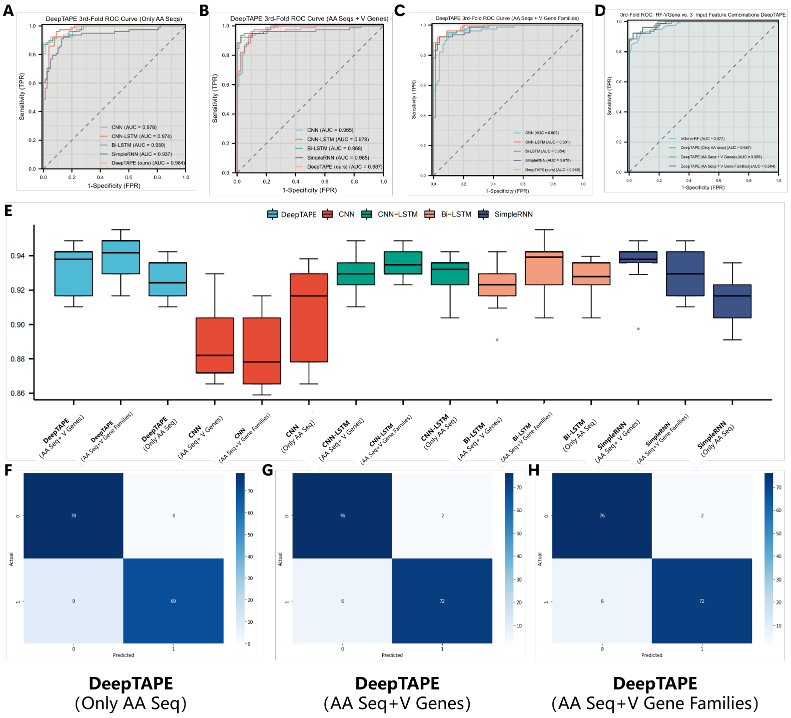

Figure S1: **Repertoire classification performance of TCRβ CDR3 amino acid sequences and gene frequencies in SLE.** (A) The ROC curve of DeepTAPE only input AA seqs, which performs better than other models with the same features. (B) The ROC curve of DeepTAPE using the combination of AA seq and V-genes, which performs better than other models with the same features. (C) The ROC curve of DeepTAPE using the combination of AA seq and V-gene families performs better than other models with the same features. (D) The ROC curves of the DeepTAPE on three input combinations, which use different features and perform better than the comparison RF-VGene. (E) Box plots comparing the performance of DeepTAPE and other models under various feature combinations. (F) Confusion matrix of DeepTAPE applied with only AA Seq features. (G) Confusion matrix of DeepTAPE applied with the combination of AA Seq and V Genes. (H) Confusion matrix of DeepTAPE applied with the combination of AA Seq and V Gene families. ROC, receiver operator curve; AA seq, amino acid sequence.

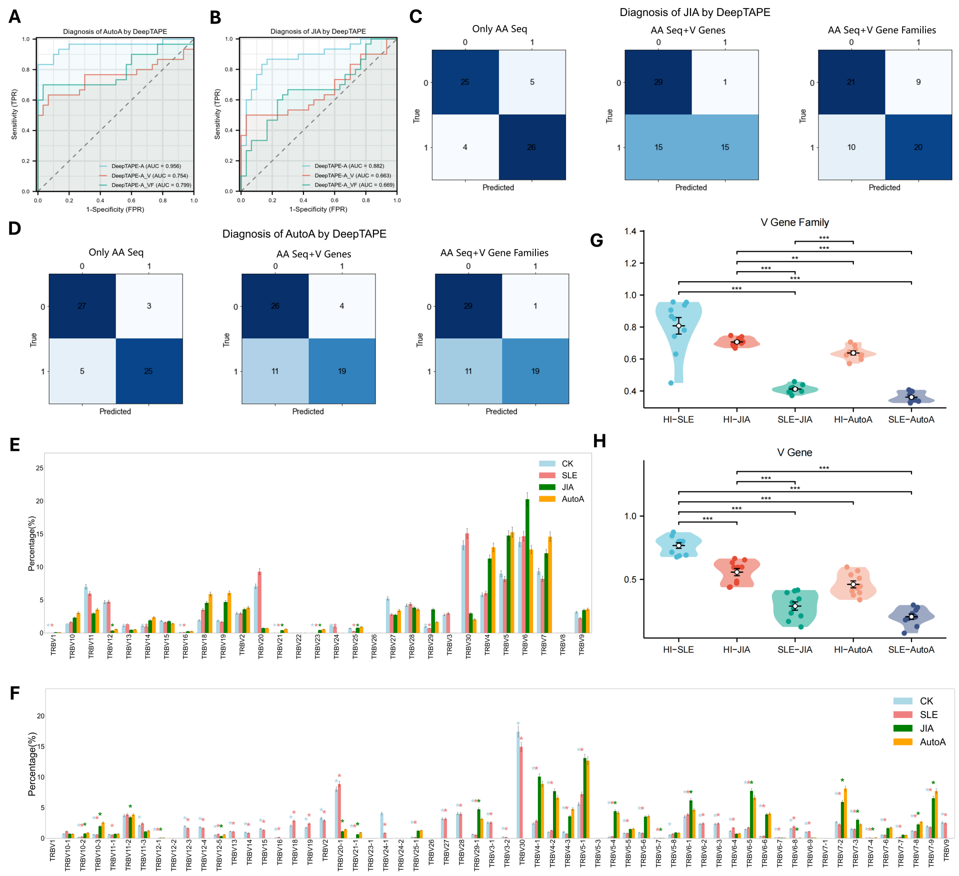

Figure S2: **Independent testing performance of the TCRβ CDR3 classifier on external autoimmune disease datasets and analysis of V gene usage differences across diseases.** (A) ROC curves for the three DeepTAPE versions in the JIA classification task, with the amino-acid-only model showing the best performance. (B) ROC curves for the three DeepTAPE versions in the AutoA classification task, with the amino-acid-only model showing the best performance. (C) Heatmaps of the confusion matrices for the three DeepTAPE versions in the JIA classification task. (D) Heatmaps of the confusion matrices for the three DeepTAPE versions in the AutoA classification task. (E) Clustered bar chart showing significant frequency differences in the V gene family of CDR3s among SLE, HI, JIA, and AutoA samples. (F) Clustered bar chart showing significant frequency differences in the V genes of CDR3s among SLE, HI, JIA, and AutoA samples. (G) Violin plot comparing the distributions of Pearson correlation coefficients of V gene family frequencies between JIA and SLE, JIA and HI, and between AutoA and SLE, AutoA and HI. The correlations with HI are significantly stronger for both JIA and AutoA (independent samples t-test, $p < 0.001$). (H) Violin plot comparing the distributions of Pearson correlation coefficients of V gene frequencies between JIA and SLE, JIA and HI, and between AutoA and SLE, AutoA and HI. The correlations with HI are significantly stronger for both JIA and AutoA (independent samples t-test, $p < 0.001$).

A

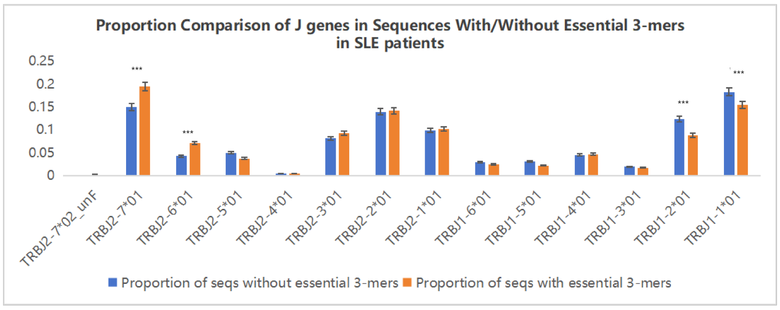

B

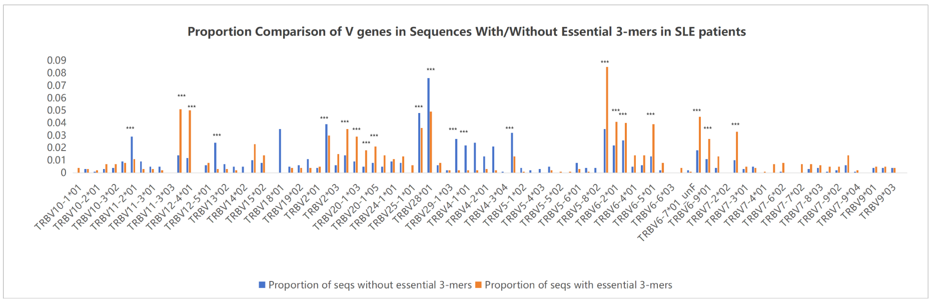

Figure S3: **Proportion comparison of TRBJ and TRBV usage in sequences with and without essential 3-mer oligopeptides in SLE patients.** (A) Proportion of CDR3β sequences assigned to each TRBJ allele among sequences without (blue) and with (orange) essential 3-mers (LFF, AFF, YTF, IYF). Error bars denote SE across patients. *** adjusted $p < 0.001$. (B) Proportion of CDR3β sequences assigned to each TRBV allele among sequences without (blue) and with (orange) essential 3-mers. Error bars denote SE across patients. *** adjusted $p < 0.001$.

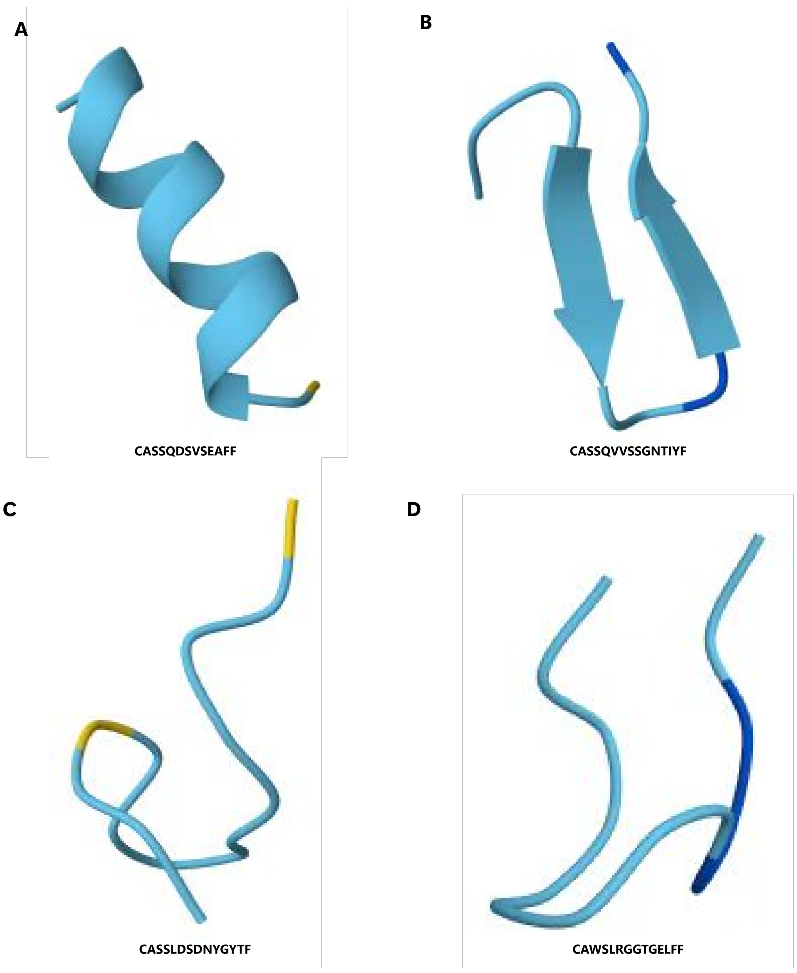

Figure S4: **AlphaFold-predicted structures of sequences containing essential 3-mer oligopeptides.** (A) Predicted structure of CASSQDSVSEAFF. (B) Predicted structure of CASSQVVSSGNTIYF. (C) Predicted structure of TCASSLDSDNYGYTF. (D) Predicted structure of CAWSLRGGTGELFF.

5 SLE Patients Clinical Information Summary

Table S5: Demographic characteristics of main dataset

Group	Age	Sex
SLE	34.5 ± 11.9	Female: 793 (91.3%) / Male: 76 (8.7%)
Control	28.9 ± 6.1	Female: 222 (50.6%) / Male: 217 (49.4%)

This table details the characteristics for the subset of samples (n = 869 for SLE patients, n = 439 for HI) with complete metadata.

Table S6: Disease status summary

Disease State	Number of Patients	Percentage (%)	Clinical Significance
Active	558	64.2	Disease is active
Silent	311	35.8	Disease is inactive or in remission

Table S7: Organ damage status summary

Damage Status	Number of Patients	Percentage (%)	Clinical Significance
One	312	35.9	Single organ damage
Two	227	26.1	Two organs damaged
Three	131	15.1	Three organs damaged
Four	18	2.1	Four organs damaged
NULL	181	20.8	No organ damage record

References

[1] Liu, X. *et al.* T cell receptor β repertoires as novel diagnostic markers for systemic lupus erythematosus and rheumatoid arthritis. *Annals of the rheumatic diseases* **78**, 1070–1078 (2019).

[2] Touil, H., Mounts, K. & De Jager, P. L. Differential impact of environmental factors on systemic and localized autoimmunity. *Frontiers in immunology* **14**, 1147447 (2023).

[3] Ramos, P. S. *et al.* A comprehensive analysis of shared loci between systemic lupus erythematosus (sle) and sixteen autoimmune diseases reveals limited genetic overlap. *PLoS genetics* **7**, e1002406 (2011).

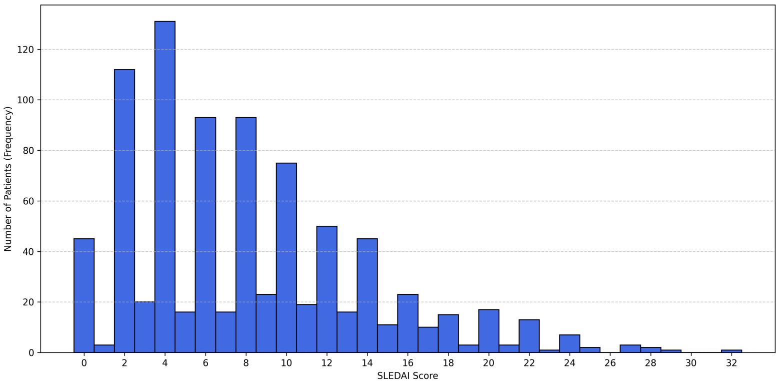

Figure S5: Distribution of SLEDAI in SLE patients

[4] Henderson, L. A. *et al.* Next-generation sequencing reveals restriction and clonotypic expansion of treg cells in juvenile idiopathic arthritis. *Arthritis & rheumatology* **68**, 1758–1768 (2016).

[5] Bonami, R. H. *et al.* Bruton's tyrosine kinase supports gut mucosal immunity and commensal microbiome recognition in autoimmune arthritis. *Frontiers in immunology* **13**, 748284 (2022).

[6] Musters, A. *et al.* In rheumatoid arthritis, synovitis at different inflammatory sites is dominated by shared but patient-specific t cell clones. *The Journal of Immunology* **201**, 417–422 (2018).

[7] Zecevic, L. *et al.* Potential immune biomarkers in diagnosis and clinical management for systemic lupus erythematosus. *Journal of Medical Biochemistry* **37**, 163 (2018).

[8] Wong, C. & Li, B. Autocat: automated cancer-associated tcrs discovery from tcr-seq data. *Bioinformatics* **38**, 589–591 (2022).

[9] Lee, L. W. *et al.* Characterisation of t cell receptor repertoires in coeliac disease. *Journal of Clinical Pathology* **77**, 116–124 (2024).

[10] Mitchell, A. M. *et al.* Temporal development of t cell receptor repertoires during childhood in health and disease. *JCI insight* **7** (2022).

[11] Lee, B. *et al.* Distinct immune characteristics distinguish hereditary and idiopathic chronic pancreatitis. *The Journal of clinical investigation* **130**, 2705–2711 (2020).

[12] Ramien, C. *et al.* T cell repertoire dynamics during pregnancy in multiple sclerosis. *Cell reports* **29**, 810–815 (2019).

[13] Rigi, A. *et al.* Ai and deep learning in understanding the etiology and pathogenesis of autoimmune diseases. *Kindle* **4**, 1–182 (2024).

[14] Mustelin, T., Bottini, N. & Stanford, S. M. The contribution of ptpn22 to rheumatic disease. *Arthritis & rheumatology* **71**, 486–495 (2019).

[15] Higgs, B. W. *et al.* Patients with systemic lupus erythematosus, myositis, rheumatoid arthritis and scleroderma share activation of a common type i interferon pathway. *Annals of the rheumatic diseases* **70**, 2029–2036 (2011).

[16] Zhang, H., Zhan, X. & Li, B. Giana allows computationally-efficient tcr clustering and multi-disease repertoire classification by isometric transformation. *Nature communications* **12**, 4699 (2021).
